# C4.4A gene ablation is compatible with normal epidermal development and causes modest overt phenotypes

**DOI:** 10.1038/srep25833

**Published:** 2016-05-12

**Authors:** Mette Camilla Kriegbaum, Benedikte Jacobsen, Annette Füchtbauer, Gert Helge Hansen, Ib Jarle Christensen, Carsten Friis Rundsten, Morten Persson, Lars Henning Engelholm, Andreas Nygaard Madsen, Ivano Di Meo, Ida Katrine Lund, Birgitte Holst, Andreas Kjaer, Ole Didrik Lærum, Ernst-Martin Füchtbauer, Michael Ploug

**Affiliations:** 1 The Finsen Laboratory, Rigshospitalet, Copenhagen, Denmark; 2 Biotech Research and Innovation Centre (BRIC), University of Copenhagen, Copenhagen, Denmark; 3 Department of Molecular Biology and Genetics, Aarhus University, Aarhus, Denmark; 4 Department of Cellular and Molecular Medicine, University of Copenhagen, Copenhagen, Denmark; 5 Department of Clinical Physiology, Nuclear Medicine & PET and Cluster for Molecular Imaging, Rigshospitalet and University of Copenhagen, Copenhagen, Denmark; 6 Deparment of Neuroscience and Pharmacology, University of Copenhagen, Denmark; 7 Unit of Molecular Neurogenetics, Foundation IRCCS Neurological Institute “Carlo Besta”, Milano, Italy; 8 Department of Pathology, Haukeland University Hospital, Bergen, Norway; 9 Department of Clinical Medicine, The Gade Laboratory of Pathology, University of Bergen, Norway

## Abstract

C4.4A is a modular glycolipid-anchored Ly6/uPAR/α-neurotoxin multidomain protein that exhibits a prominent membrane-associated expression in stratified squamous epithelia. C4.4A is also expressed in various solid cancer lesions, where high expression levels often are correlated to poor prognosis. Circumstantial evidence suggests a role for C4.4A in cell adhesion, migration, and invasion, but a well-defined biological function is currently unknown. In the present study, we have generated and characterized the first C4.4A-deficient mouse line to gain insight into the functional significance of C4.4A in normal physiology and cancer progression. The unchallenged C4.4A-deficient mice were viable, fertile, born in a normal Mendelian distribution and, surprisingly, displayed normal development of squamous epithelia. The C4.4A-deficient mice were, nonetheless, significantly lighter than littermate controls predominantly due to differences in fat mass. Congenital C4.4A deficiency delayed migration of keratinocytes enclosing incisional skin wounds in male mice. In chemically induced bladder carcinomas, C4.4A deficiency attenuated the incidence of invasive lesions despite having no effect on total tumour burden. This new C4.4A-deficient mouse line provides a useful platform for future studies on functional aspects of C4.4A in tumour cell invasion *in vivo.*

The skin is a highly complex organ forming an important first-line protective and mechanical barrier against water loss and opportunistic pathogens. The outer lining of the skin, the epidermis, is comprised of keratinocytes arranged in a stratified squamous epithelium, which includes a proliferative *stratum basale* giving rise to the differentiating suprabasal layers, stratified into *stratum spinosum*, *stratum granulosum* and finally *stratum corneum*^1^. Not only the skin but also other organs such as the esophagus and vagina are lined by stratified squamous epithelia. The structural and functional integrity of these epithelia depend on highly complex molecular interactions between cytoskeletal components, specific cell and matrix adhesion complexes, as well as defined ion channels^1^. Congenital, autoimmune or pathogen-induced perturbation of this intricate communication often leads to severe disorders^2^,^3^,^4^.

A newly recognized and highly regulated abundant component of the squamous epithelium is the modular C4.4A protein, which is a glycolipid-anchored multidomain member of the Ly6/uPAR/α-neurotoxin (LU) protein domain family^5^. Another member of this protein family expressed in keratinocytes is SLURP1, which upon congenital deficiency causes palmoplantar keratoderma in both humans (a disorder known as Mal de Meleda) and in genetic mouse models^6^,^7^,^8^. In humans and rodents, C4.4A expression is primarily confined to the plasma membrane of cells residing in the suprabasal layer of the epithelium, primarily the *stratum spinosum*, thus leaving *stratum basale* devoid of C4.4A expression^9^,^10^,^11^. As revealed by a global protein expression survey in unchallenged mice and rats, high C4.4A expression is found in all squamous epithelia including skin, oral cavity, esophagus, cornea, vagina and non-glandular part of the rodent stomach^11^. An all or none appearance of C4.4A expression at squamo–columnar transition zones, *e.g.* the utero–vaginal junction, and its emergence at embryonic day 13.5 in the nasal cavity coinciding with the development of *stratum spinosum* argue for a strict gene regulation during embryonic development and during homeostasis at adulthood. Accordingly, gene transcription of C4.4A (*Lypd3*) is regulated by C/EBPβ, which is a downstream mediator of Notch signalling, controlling the switch to the spinous cell fate^12^,^13^. C4.4A is thus a biomarker of early squamous differentiation.

Historically, C4.4A was selected and defined as a metastasis-associated protein due to its expression in an aggressive metastasizing rat pancreatic adenocarcinoma cell line compared to a corresponding non-metastasizing counterpart^14^. Prompted by the alleged association to cancer metastasis, a number of independent studies subsequently reported the expression of C4.4A in several different cancer lesions, including solid carcinomas of various origins such as bladder, lung, esophagus, skin, pancreas, colon and stomach^9^,^10^,^15^,^16^,^17^,^18^,^19^,^20^,^21^,^22^. Intriguingly, the majority of these studies reported a significant correlation between high C4.4A expression levels in the primary tumour lesions and poor survival of patients suffering from *e.g.* lung adenocarcinomas, gastric cancer, colorectal cancer and esophageal cancer^15^,^16^,^17^,^18^,^19^. A functional cause relationship for this correlation remains nonetheless to be explored. Circumstantial evidence suggests that C4.4A could play a role in adhesion, migration and invasion since *LYPD3* transcription is inducible in human urothelial cells upon exposure to extracellular matrix proteins^20^ and C4.4A-positive, but not C4.4A-negative rat pancreatic tumour cells, are capable of transmigrating Matrigel-coated transwell plates^22^. In line with this, C4.4A expression is induced in the invasive front of esophageal squamous cell carcinoma (SCC), colorectal cancer as well as SCC of the skin^9^,^10^,^18^.

To enable controlled studies on the impact of C4.4A expression in normal physiology as well as pathophysiology *in vivo*, we have generated a C4.4A-deficient mouse line and characterized it for overt and challenged-induced phenotypes. The overt phenotypes presented by the C4.4A-deficient mice include a clear and consistent attenuation in growth related to adipose tissue but, surprisingly, development of the various squamous epithelia appeared normal and no gross functional abnormalities were evident.

## Results

### C4.4A-deficient mice are lighter than littermate controls

**Figure 1:**
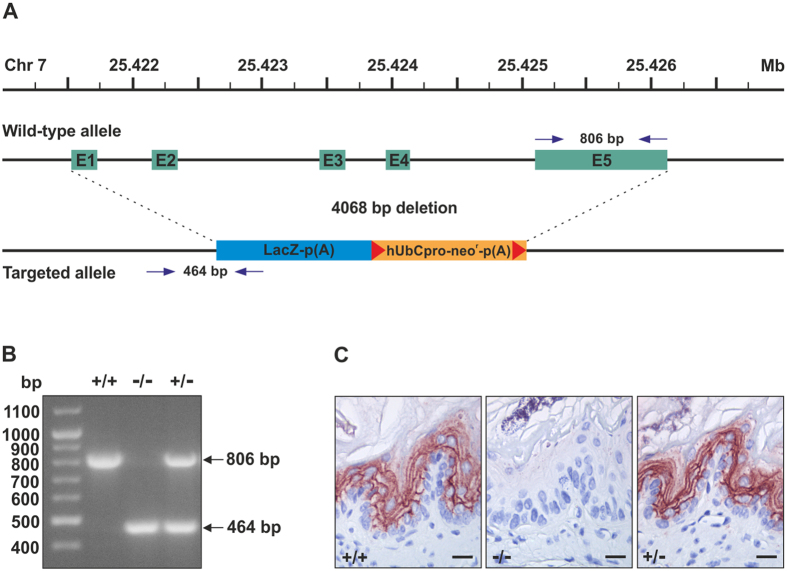
Global deletion of *Lypd3*. Panel (**A**) shows an illustration of the targeting strategy. The wild-type *Lypd3* allele (top) is a 4549 bp gene located on chromosome 7 (position 25,421,589–25,426,137). The targeted *Lypd3* allele (bottom) has a 4068 bp deletion of exon 1–5 at position 25,421,612–25,425,679 by replacement with a β-galactosidase-neomycin cassette by homologous recombination. The gene positions on the chromosome are referring to the NCBI mouse genome build 37. Panel (**B**) shows the PCR fragments after genotyping of C4.4A^+/+^, C4.4A^−/−^ and C4.4A^+/−^ mice. The expected PCR fragments from wild-type (806 bp) and C4.4A-deficient (464 bp) mice are indicated by arrows. The position of the used primer pairs (arrows) and the PCR fragment lengths are also indicated in panel (**A**). Panel (**C**) shows staining of C4.4A^+/+^, C4.4A^−/−^ and C4.4A^+/−^ formalin-fixed and paraffin-embedded esophagus using a rabbit anti-C4.4A pAb. Abbreviations and symbols: E: exon; LacZ: β-galactosidase coding sequence from the *E. coli* lacZ gene; hUbCpro: promoter from the human ubiquitin C gene; neo^r^: coding sequence for neomycin phosphotransferase; p(A): polyadenylation signal; red triangles: loxP sites. Scale bars = 25 μm.

C4.4A-deficient mice were generated from *Lypd3*-targeted embryonic stem (ES) cells, in which exons 1 to 5 of the *Lypd3* allele were replaced by a β-galactosidase reporter gene and a neomycin resistance cassette (Fig. 1A,B). This deleted the translation start site and the main part of the C4.4A coding sequence, resulting in a complete termination in biosynthesis of C4.4A at the protein level as illustrated by immunohistochemical staining of esophagus (Fig. 1C).

**Figure 2:**
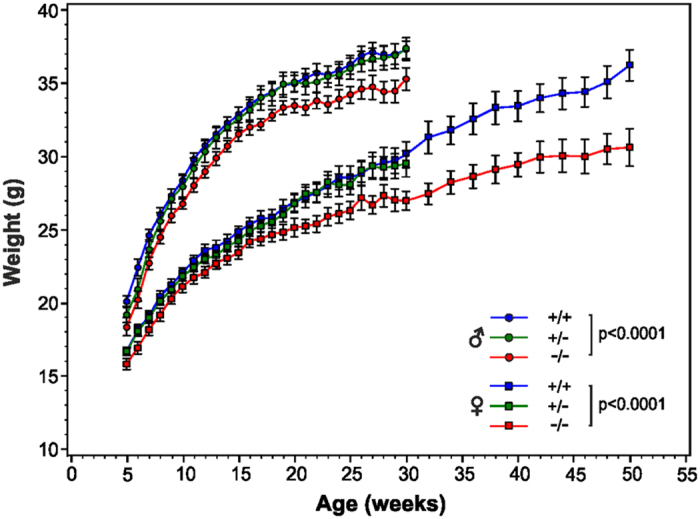
C4.4A-deficient mice are lighter than littermate controls. A cohort of F2 female C4.4A^−/−^ (n = 14), C4.4A^+/−^ (n = 15) and C4.4A^+/+^ (n = 16) mice as well as male C4.4A^−/−^ (n = 11), C4.4A^+/−^ (n = 14) and C4.4A^+/+^ (n = 14) mice were weighed every week from 5–30 weeks of age. At 30 weeks of age, all males as well as all C4.4A^+/−^ female mice were sacrificed. 14 C4.4A^+/+^ and 14 C4.4A^−/−^ female mice were weighed every second week for an additional 20 weeks until 50 weeks of age. Both female and male C4.4A-deficient mice were significantly lighter than littermate controls. The growth curves of the three genotypes for each gender were compared with log-transformed data using mixed modelling statistics for longitudinal effects. Standard errors of the mean are shown.

To examine the effect of C4.4A deficiency, we mated C4.4A^+/−^ F1 C57BL/6 mice to generate C4.4A^−/−^, C4.4A^+/−^ and C4.4A^+/+^ littermates. Analysis of the weaned F2 offspring revealed a normal Mendelian distribution (Supplementary table S1). To obtain insight into the survival and development of C4.4A-deficient mice, a prospective cohort of F2 C4.4A^−/−^, C4.4A^+/−^ and C4.4A^+/+^ littermate male and female mice were observed and weighed every week from 5 to 30 weeks of age. The C4.4A^−/−^ mice appeared macroscopically normal, had normal survival and were fertile. Nonetheless, C4.4A^−/−^ mice were significantly lighter than both C4.4A^+/+^ and C4.4A^+/−^ littermates and this applied to both genders (Fig. 2). The weight difference was essentially sustained from 5 to 30 weeks of age, with the males being 5.8% (p < 0.0001) lighter and females being 6.4% (p < 0.0001) lighter than their wild-type littermates as analysed on log-transformed data using mixed modelling. The weight difference did, however, progressively increase with age in the females, as manifested by the greater separation of the weight curves. The C4.4A^−/−^ and C4.4A^+/+^ female mice were followed for another 20 weeks until 50 weeks of age, at which the weight difference had increased to 15.9% (p < 0.0001). No mice died in either group during the observation period indicating no major difference in life spans. The coefficient of variation (CV) for the weight data was 11% for all mice observed between 5 and 50 weeks independent of genotype, gender and age.

To investigate if the difference in weight was present already at birth, a new cohort of F3 mice was weighed from the day of birth (day 0) until 6 weeks of age (Supplementary Fig. S1). Reassuringly, the significant weight difference was recapitulated for both genders by this new cohort of mice (female C4.4A^+/+^ vs. C4.4A^−/−^: p < 0.0001, male C4.4A^+/+^ vs. C4.4A^−/−^: p = 0.018). Notably, at birth, the weight of the female C4.4A^−/−^ and C4.4A^+/+^ pups was similar (p = 0.74), and the weight difference became significant at 2 weeks of age (p = 0.002), indicating that the induction in weight phenotype occurred postnatal. In the case of male mice, significance in weight difference could only be obtained for the continuous data set indicating that either the onset of the weight phenotype is delayed or the impact is less pronounced to reach significance at these early time points. For all mice observed between 0 and 6 weeks, the CV was 21% independent of genotype, gender and age.

### C4.4A-deficient mice are leaner than littermate controls

**Figure 3:**
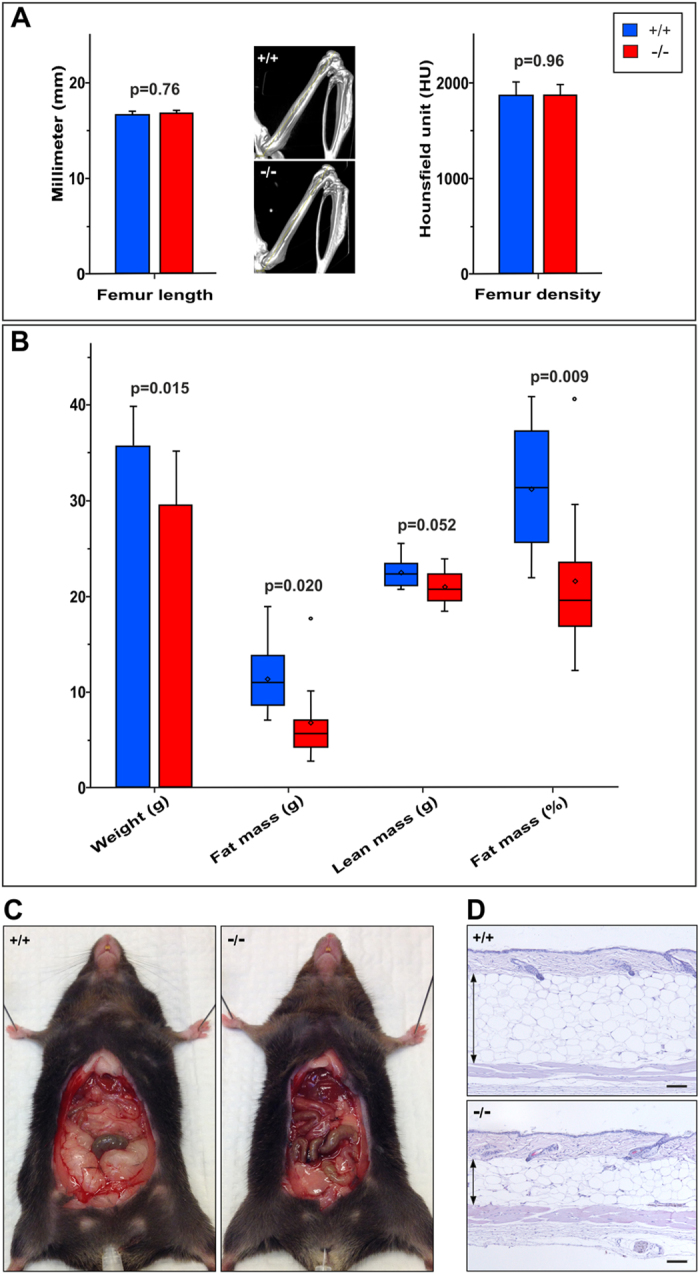
C4.4A-deficient mice are leaner than littermate controls. The 52 weeks old female F2 cohort C4.4A^+/+^ (n = 10) and C4.4A^−/−^ (n = 10) mice were analysed by CT scanning and MRI to determine their bone lengths, bone mineral densities and body compositions, respectively. The lengths and densities of the femurs were measured on reconstructed CT data using the Inveon visualization software and revealed no difference between genotypes (**A**). Representative CT-scans of femurs from C4.4A^+/+^ and C4.4A^−/−^ mice are shown. The MRI data were used to determine the total fat and lean masses and calculate the fat mass in percentage of total body weight, here presented as boxplots (**B**). The mean estimates in fat mass percentages were 31.3% (C4.4A^+/+^) and 21.6% (C4.4A^−/−^). The difference in fat mass was both due to differences in abdominal fat mass (**C**) and subcutaneous fat (**D**). The shown C4.4A^+/+^ and C4.4A^−/−^ mice in panel (**C**) had 37.2% and 13.2% body fat, respectively. The H&E staining in panel D are from C4.4A^+/+^ and C4.4A^−/−^ mice with 38.4% and 20.7% fat mass, respectively. The means and medians in panel (**B**) are indicated by squares and horizontal lines, respectively. The arrows in panel (**D**) indicate the thickness of the subcutaneous fat. Standard deviations are shown. Scale bars = 100 μm.

To disclose whether the observed weight difference between C4.4A^−/−^ and C4.4A^+/+^ mice reflects differences in bone growth and/or body composition, we randomly included 10 mice of each genotype from the 52 weeks old F2 cohort female mice for examination by computed tomography (CT) and magnetic resonance imaging (MRI). Data from CT scans revealed that C4.4A^−/−^ and C4.4A^+/+^ mice had equal bone lengths (p = 0.76, Fig. 3A) and similar bone densities (p = 0.96, Fig.3A). As opposed to this, the MRI showed a significant difference in the body composition between genotypes (Fig. 3B). Total fat mass was significantly reduced in C4.4A^−/−^ mice (p = 0.020), whereas difference in total lean mass was borderline significant (p = 0.052). C4.4A^−/−^ mice were on average 9.7% (p = 0.009) less fat than C4.4A^+/+^ mice (21.6% vs 31.3%), when comparing the calculated body fat mass in percentage of the total body weight. This difference in fat mass could be assigned to both reduced abdominal fat (Fig. 3C) and subcutaneous fat (Fig. 3D), as illustrated by the pictures of mice representing extremes in the recorded cohort.

In conclusion, these data show that the observed weight difference primarily could be ascribed to a reduced fat deposition in the C4.4A^−/−^ mice and not to skeletal size or bone mineral density.

### C4.4A-deficient mice have normal squamous epithelia morphology and exhibit normal transepidermal water loss

**Figure 4:**
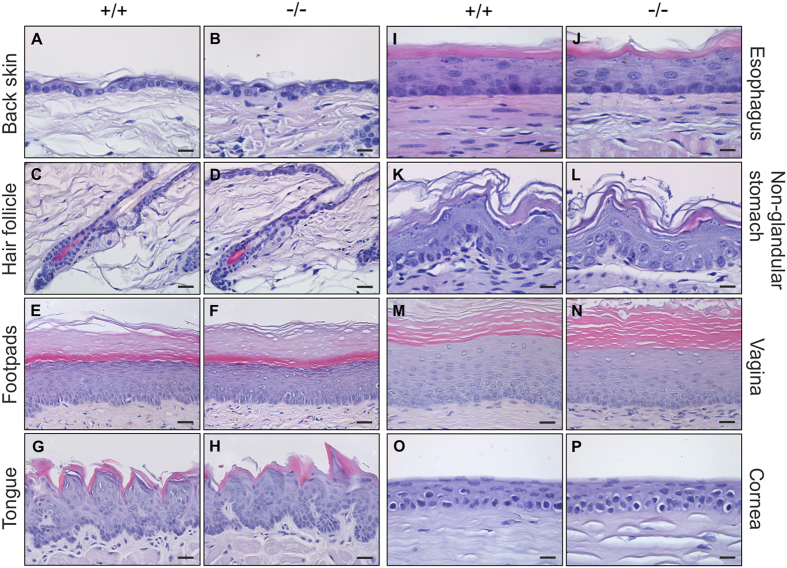
52 weeks old C4.4A-deficient female mice have normal appearing squamous epithelia. Tissues from 52 weeks old F2 female mice were formalin-fixed, paraffin-embedded, sectioned and stained by H&E for histological analysis. The examined tissues are back skin (**A**,**B**), hair follicles (**C**,**D**), footpads (**E**,**F**), tongue (**G**,**H**), esophagus (**I**,**J**), non-glandular stomach (**K**,**L**), vagina (**M**,**N**) and cornea of the eye (**O**,**P**). C4.4A-deficient mice revealed no obvious histological differences from wild-type littermates. Scale bars = A&B, I–L, O&P (15 μm); C–H, M&N (25 μm).

Under normal homeostatic conditions, C4.4A expression is primarily confined to the suprabasal layers of stratified squamous epithelia^11^. We therefore compared the morphology of unchallenged squamous epithelia between C4.4A^−/−^ and the corresponding C4.4A^+/+^ littermates at various locations in young (1, 2 and 3 weeks old), adult (7, 8, 12 and 13 weeks old) and old (30 and 52 weeks old) mice. No profound pathological changes were found, as illustrated by haematoxylin and eosin (H&E) staining of back skin, hair follicles, footpads, tongue, esophagus, non-glandular stomach, vagina and cornea of the eye from 52 weeks old female mice (Fig. 4). Since C4.4A is a marker of squamous differentiation, we also analysed if the conventional markers of differentiation, *i.e.* cytokeratin (CK) 14 (*stratum basale*), CK10 (suprabasal layers), loricrin (*stratum granulosum* and *stratum corneum*) as well as the structural homologue Haldisin^23^ (*stratum granulosum*) were dysregulated in back skin of young, adult and old C4.4A^−/−^ mice. C4.4A deficiency did not result in obvious differences in the expression of these epidermal differentiation markers, when analysed by immunohistochemistry (data not shown).

**Figure 5:**
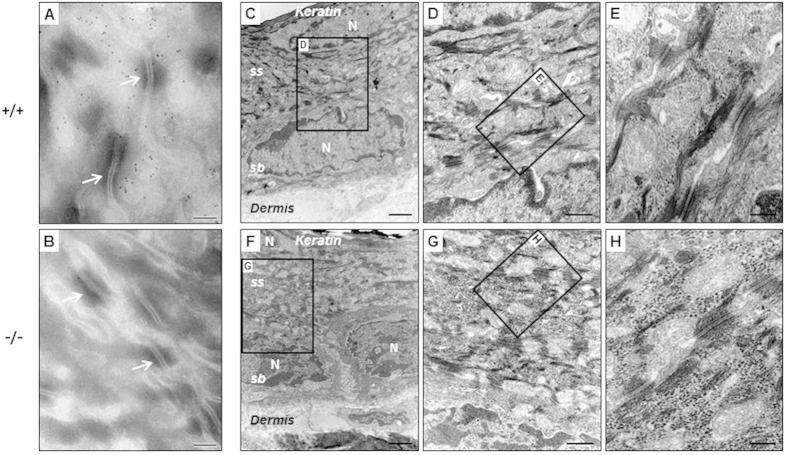
Subcellular localization of C4.4A and analysis of back skin from C4.4A-deficient mice by transmission electron microscopy. Panels (**A**) and (**B**) show pictures of immunogold labelling of C4.4A performed on ultracryosections of footpads from 6 weeks old C4.4A^+/+^ (**A**) and C4.4A^−/−^ (**B**) littermates using a rabbit anti-C4.4A pAb. C4.4A was localized in the cell membrane and was excluded from the desmosomes (indicated by arrows). Panels (**C**–**H)** show low and high magnification pictures of Epon-embedded back skin from 10 weeks old male C4.4A^+/+^ (**C**–**E**) and C4.4A^−/−^ (**F**–**H**) mice. No obvious overt membrane phenotypes were revealed at the subcellular level. Abbreviations: *sb*: *stratum basale*; *ss*: *stratum spinosum*; N: nucleus. Scale bars = A, B, (**E**,**H**) (0.2 μm); (**D**,**G**) (0.5 μm); (**C**,**F**) (2 μm).

Further, we examined if any ultrastructural abnormalities were present in the C4.4A^−/−^ mice by transmission electron microscopy. Initially, the subcellular localization of C4.4A was visualized by immunogold labelling on cryosections from mouse footpads. As expected, C4.4A was found associated to the cell membrane, but it was specifically excluded from the desmosomes bridging the keratinocytes (Fig. 5A). As an internal control for specificity, C4.4A^−/−^ footpads were analysed in parallel (Fig. 5B). Epon-embedded tissues from littermate C4.4A^−/−^ and C4.4A^+/+^ male and female mice containing squamous epithelia (back skin, ear skin, footpads and non-glandular stomach) were subsequently processed and examined by transmission electron microscopy. At the subcellular level no consistent abnormalities were detected in the C4.4A^−/−^ mice as compared to wild-type littermates (Fig. 5C–H).

If subtle functional defects in the skin barrier were introduced by C4.4A deficiency, a small, sustained transepidermal water loss (TEWL) might arise despite the lack in obvious histological epidermal phenotypes. This could further increase energy metabolism and thus attenuate weight gain. To challenge this potential relationship, we explored the impact of C4.4A on maintenance of the skin barrier integrity by measuring TEWL in newborn pups. The kinetics of water loss over time, as calculated by weight loss, however, showed no statistically significant difference between genotypes (C4.4A^−/−^ vs. C4.4A^+/+^, p = 0.58, Supplementary Fig. S2).

### C4.4A-deficient mice display normal energy expenditure

Changes in body weights often correlate with an altered energy homeostasis. To explore whether the observed impact of the C4.4A genotype on fat mass is mirrored by significant perturbations in the energy expenditure we monitored metabolic parameters for 12–14 weeks old female and male littermate C4.4A^−/−^ and C4.4A^+/+^ mice using metabolic cages for a period of 60 hours. A modest decrease in the food intake was observed for C4.4A^−/−^ female mice compared to C4.4A^+/+^ (13.8%, p < 0.0001, Supplementary Fig. 3A), which is well aligned with the observation that these mice are slightly leaner. A similar reduction was however not observed for males, where C4.4A^−/−^ mice actually presented a negligible 1.2% increase in food consumption compared to C4.4A^+/+^ (p = 0.03). None or only very subtle differences were observed in physical activity, energy expenditure, or respiratory quotient (vol CO_2_/vol O_2_) between genotypes of the same gender (Supplementary Fig. S3B–D). To reveal if a small genetic predisposition to energy dissipation nevertheless exists in the C4.4A-deficient mice, we also measured the mRNA expression levels of master genes involved in adipogenesis (*C/ebpβ* and *Pparγ*) and adaptive thermogenesis via *Ucp1*, which mediates uncoupling of oxidative phosphorylation in mithochondria^24^. We found no genotype-specific difference in the expression levels of these genes in neither white adipose tissue depots [retroperitoneal (rWAT) and gonadal (gWAT)] nor in brown adipose tissue depots (BAT) by qPCR (Supplementary Fig. S4).

### Few genes are dysregulated in the skin of C4.4A-deficient mice

To identify potential dysregulated genes induced by *Lypd3* gene ablation, global mRNA sequencing profiles from C4.4A^−/−^ mice and littermate control C4.4A^+/+^ mice were compared. cDNA libraries, generated from high-quality RNA (RNA integrity number of 8.10–9.20) extracted from ears, were sequenced by paired-end sequencing and aligned to the UCSC mm10 reference genome, and gene counts were compared between genotypes for males and females, respectively. All four sample types (female and male; C4.4A^−/−^ and C4.4A^+/+^) could be neatly separated as evident from the principal component analysis in Supplementary Fig. S5. Only few genes exhibited significant dysregulation in both genders (Supplementary tables S2 & S3). Genes involved in the inflammatory response tended to be slightly overrepresented in the C4.4A^−/−^ mice, albeit the magnitudes of the differences were rather modest. Notably, neighbouring genes to *Lypd3* (*Ethe1*, *Tex101, LOC102640044*, *Phldb3*, *BC049730)* were all significantly dysregulated, which most likely relates to the physical manipulation of the genome rather than a specific effect of C4.4A deficiency. Of particular relevance to the spontaneous weight difference exhibited by our C4.4A-deficient mice, one of these genes, *Ethe1*, (downregulated by app. 2.5-fold) encodes a mitochondrial sulfur dioxygenase involved in sulfide detoxification by oxidation of persulfide to sulfite. Loss-of-function mutations in *ETHE1* cause the fatal disorder ethylmalonic encephalopathy in humans^25^, and *Ethe1*-deficient mice show growth arrest from postnatal day 14^26^. To eliminate downregulation of *Ethe1* as a confounding factor in the development of overt phenotypes in our C4.4A-deficient mice, we measured the levels of thiosulfate in serum and urine as a sensitive diagnostic marker of Ethe1 dysfunction. Reassuringly, thiosulfate levels recorded for C4.4A^−/−^ and C4.4A^+/+^ mice showed no statistical differences (p = 0.75 and p = 0.97, respectively, Supplementary Fig. S6) and were all within the established normal range^27^.

### C4.4A deficiency imposes a delay in skin wound healing in male mice

**Figure 6:**
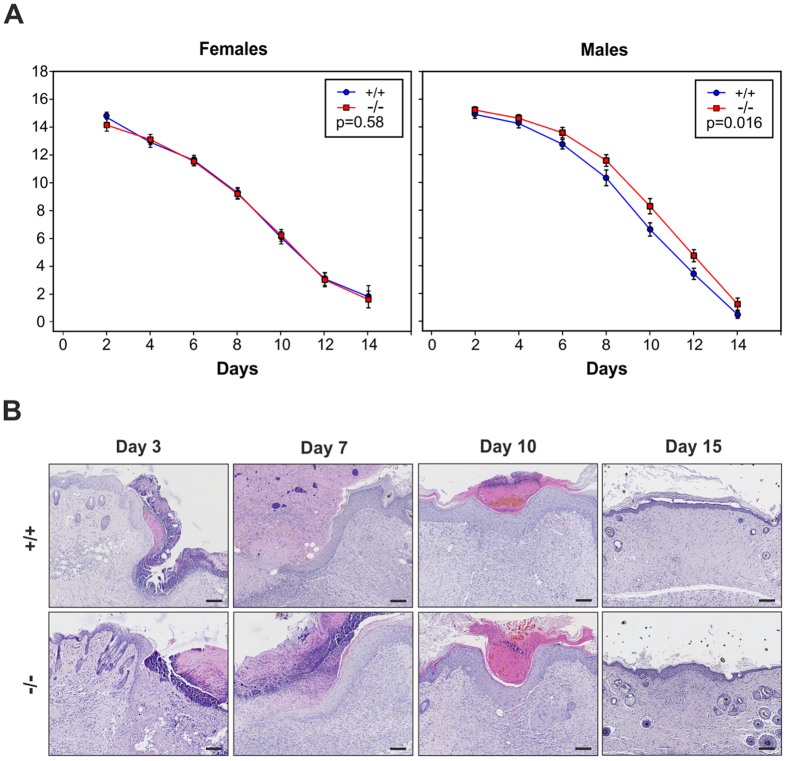
C4.4A deficiency delay skin wound healing in male mice. Incisional 20 mm back skin wounds were made, and the wound lengths were measured every second day until closure. Panel (**A**) shows the mean length of healing wounds of female C4.4A^+/+^ (n = 13) and C4.4A^−/−^ (n = 11) (left) and male C4.4A^+/+^ (n = 13) and C4.4A^−/−^ (n = 12) (right) mice over time. At day 14, the majority of the wounds were closed, and this time point therefore only contains measurements from 2 C4.4A^+/+^ and 5 C4.4A^−/−^ female mice as well as 4 C4.4A^+/+^ and 6 C4.4A^−/−^ male mice. Panel (**B**) shows the histological appearance of the C4.4A^+/+^ and C4.4A^−/−^ male skin wounds at day 3, 7, 10 post-wounding and after healing at day 15. Standard errors of the mean are shown. Scale bars = 100 μm.

Due to the pronounced expression of C4.4A in keratinocytes and its alleged role in migration *in vitro*^22^,^28^ we next explored how C4.4A deficiency impacts the kinetics of wound healing *in vivo*. Standardized 20 mm long, full-thickness, incisional skin wounds were generated on the back skin of C4.4A^−/−^ and C4.4A^+/+^ littermate mice representing both genders. The wound lengths were recorded until wound closure and revealed no significant difference between genotypes in the female mice (p = 0.58), whereas C4.4A-deficient male mice exhibited a significant albeit modest attenuation in the healing kinetics as compared to the corresponding wild-type mice (p = 0.016) (Fig. 6A). This delay, however, did not translate into evident morphological alterations between the genotypes as revealed by a histological examination of the wounds at day 3, 7, 10 and after closure at day 15 (Fig. 6B).

### Chemical induction of precursor and malignant bladder lesions in C4.4A-deficient mice

Previous *in vitro* studies have shown that exposure to extracellular matrix proteins induces C4.4A expression in human urothelial cells and that C4.4A is expressed in human urothelial cell carcinomas (UCC)^20^. As a logical extension of these findings, we examined the impact of C4.4A deficiency on bladder cancer progression *in vivo* using a chemically induced mouse bladder cancer model, in which oral administration of the carcinogen N-butyl-N-(4-hydroxybutyl)nitrosamine (BBN) in the drinking water induces precursor lesions that subsequently progress into malignant bladder cancer^29^.

**Figure 7:**
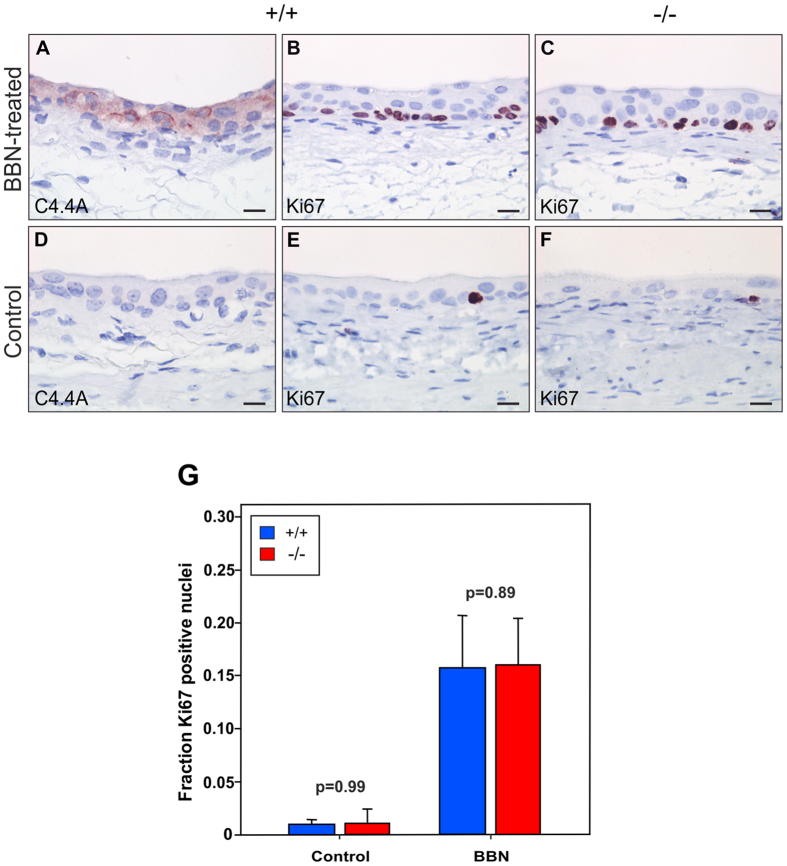
C4.4A deficiency has no impact on development of bladder precursor lesions. Female C4.4A^+/+^ (n = 9) and C4.4A^−/−^ (n = 8) mice were exposed to BBN for 4 weeks to induce urothelial hyperplasia. The C4.4A^+/+^ urothelium had cytoplasmic and membrane-bound expression of C4.4A (**A**), whereas C4.4A expression was absent in control urothelium exposed to water for 26 weeks (C4.4A^+/+^, n = 3 and C4.4A^−/−^, n = 6) (**D**). After BBN-treatment both C4.4A^+/+^ (**B**) and C4.4A^−/−^ (**C**) bladders were hyperproliferative as determined by Ki67 staining, whereas control bladders were almost Ki67-negative (**E**,**F**). Ki67-expressing nuclei as well as total nuclei in the urothelium of C4.4A^+/+^ and C4.4A^−/−^ mice were determined using the Visiomorph software. The ratio between Ki67-positive and total nuclei was calculated and showed that the number of proliferative cells was equal between genotypes (**G**). Standard deviations are shown. Scale bars = 15 μm.

To examine if C4.4A deficiency influences the induction of early reactive changes in the form of urothelial hyperplasia, we exposed female C4.4A^−/−^ and C4.4A^+/+^ mice to BBN for 4 weeks^29^. The resected bladders were examined for induction of C4.4A expression in wild-type mice by immunohistochemistry, whereas Ki67 expression served as a surrogate marker for hyperplasia in both genotypes (Fig. 7). A weak cytoplasmic and membrane-bound C4.4A expression emerged in the urothelium, predominantly in association with Ki67 expressing foci in the basal layer (Fig. 7A). The BBN-treated bladders revealed a marked upregulation of Ki67 expression in basal layers of the urothelium (Fig. 7B,C), which normally present very few Ki67-positive cells due to its exceptionally low cell turnover rate during homeostasis^30^ (Fig. 7E,F). The proliferation rates of the urothelium were, however, unaffected by the imposed C4.4A deficiency at basal conditions (p = 0.99), as well as during hyperplasia (p = 0.89) as judged by the frequency of Ki67-positive urothelial nuclei in the two genotypes (Fig. 7G).

**Figure 8:**
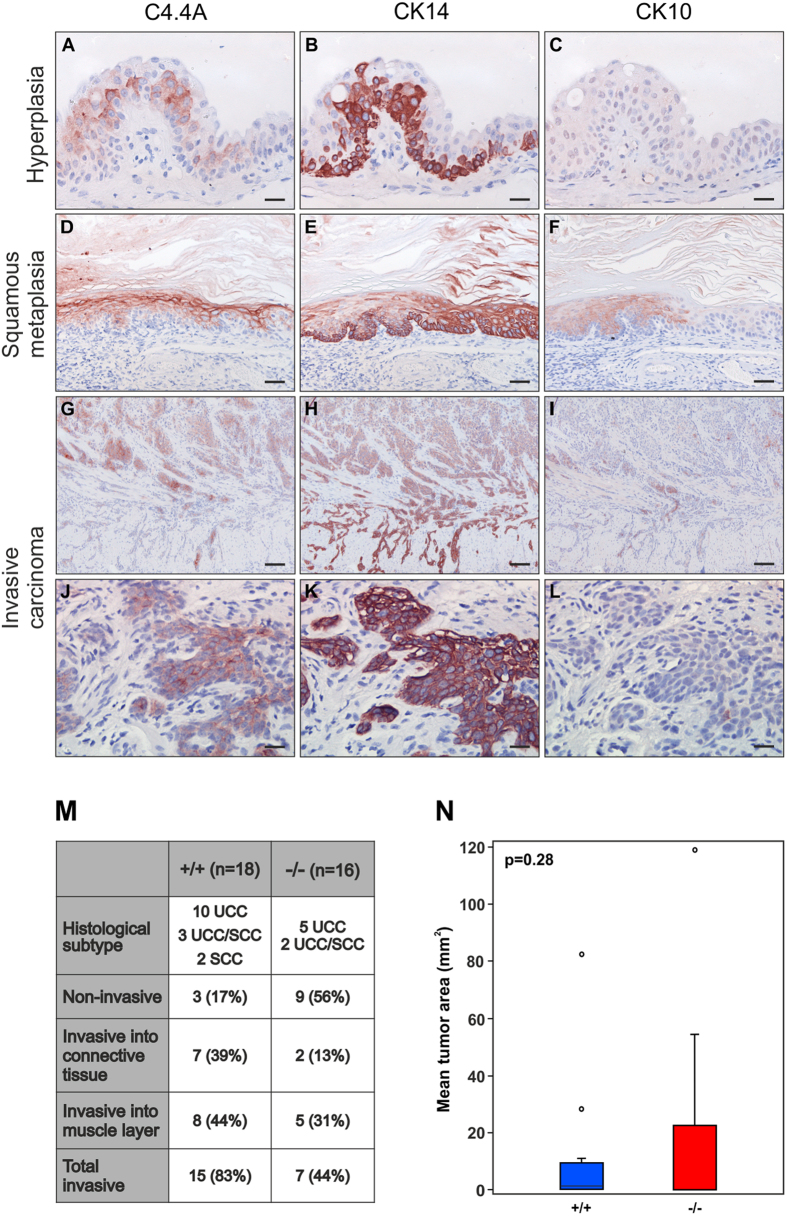
C4.4A deficiency may have an impact on development of malignant bladder lesions. Female C4.4A^+/+^ (n = 18) and C4.4A^−/−^ (n = 16) mice were exposed to BBN for 14 weeks followed by 12 weeks of water to induce malignant carcinomas. Serial sections of C4.4A^+/+^ bladders stained for C4.4A, CK14 and CK10 are shown in panels (**A–L**). C4.4A expression was induced in the hyperplastic suprabasal layers with induction of CK14 in the same cells but absence of CK10 (**A**–**C**). C4.4A expression overlaps with both CK14 and CK10 in squamous metaplasia (**D**–**F**) and invasive carcinomas (**G**–**L**). The number of mice/bladders with the given histological subtypes and invasive features were determined (**M**) and the incidence in percentage divided on genotypes was calculated as shown in brackets. The mean tumour area was determined from H&E-stained sections using the Visiomorph software (**N**). Standard deviations are shown. Scale bars = (**A**–**C**,**J**–**L**) (25 μm); (**D**–**F**) (50 μm); (**G**–**I**) (100 μm).

To study the impact of C4.4A expression on the subsequent progression into invasive bladder carcinomas, we prolonged the BBN induction time to 26 weeks^29^. The resected bladders presented a mix of morphological changes ranging from precursor lesions (hyperplasia, dysplasia, squamous metaplasia) to manifest invasive carcinomas. In the precursor lesions, C4.4A was heterogeneously expressed in the suprabasal layers of the hyperplastic urothelium of C4.4A^+/+^ mice, being primarily localized to the lower layers, indicative of *de novo* synthesis of C4.4A in cells derived from the basal layer (Fig. 8A). Interestingly, C4.4A-expressing foci were associated to suprabasal CK14 expression, whereas CK14 was restricted to the basal layer in the less transformed urothelium (Fig. 8B). CK10 expression was absent in hyperplastic urothelium (Fig. 8C). Squamous metaplasia of the urothelium always led to a translocation of the C4.4A expression into a predominantly membrane-associated state, and this overlapped with CK10 (appearing with squamous alterations) and CK14 expression (Fig. 8D–F).

In the invasive tumours, C4.4A was heterogeneously expressed (Fig. 8G,J) and was induced in both histological subtypes developed in this model, namely SCC and UCC. When C4.4A was expressed by invasive strands of tumour cells, these were generally surrounded by a single layer of C4.4A-negative “basal-like” tumour cells (Fig. 8J). This architecture recapitulates the histology observed in other invasive human carcinomas^9^,^10^.

Both C4.4A^−/−^ and C4.4A^+/+^ mice developed a mixture of invasive SCC and UCC upon the prolonged exposure to BBN (Fig. 8M). Interestingly, C4.4A^−/−^ mice developed fewer invasive tumours (44% of the mice) compared to the corresponding C4.4A^+/+^ littermate controls (83% of the mice). This difference was primarily due to lesions invading into the connective tissue (C4.4A^−/−^; 13% and C4.4A^+/+^; 39%) (Fig. 8M). Despite this, we could detect no statistically significant difference between genotypes in the tumour burden as determined by measuring the tumour area on H&E-stained sections using the Visiomorph software (p = 0.28) (Fig. 8N).

### Subcutaneous transplants of Lewis Lung cells in C4.4A-deficient mice

Finally, we tested if the stromal expression of C4.4A affected tumour take and tumour growth of a subcutaneous engraftment of Lewis Lung carcinoma cells. In this experiment, littermate C4.4A^−/−^ and C4.4A^+/+^ mice received a subcutaneous engraftment of 2.5 × 10^6^ Lewis Lung carcinoma cells. Tumour take was 100% in both genotypes and the tumour volume of the engraftment expanded with similar kinetics (p = 0.36) as measured until day 12, where all mice were sacrificed due to ethical considerations (Supplementary Fig. S7).

## Discussion

C4.4A is a robust biomarker for squamous differentiation^11^ and is allegedly involved in cell adhesion, migration, invasion and metastasis^14^,^20^,^22^,^28^. Despite these implications, a direct causative role of C4.4A has yet to be demonstrated *in vivo*. Pertaining to this, we have in the present study generated and characterized a new C4.4A-deficient mouse line, enabling controlled investigation of the functional impact of C4.4A during development and maintenance of normal homeostatic squamous epithelia as well as in cancer progression. C4.4A expression is induced late during mouse embryogenesis with the first appearance of *stratum spinosum* in the nasal cavity at day E13.5^11^. Consequently, we did not expect the imposed C4.4A deficiency to severely impact early embryonic development. Our present data clearly support this proposition, as the C4.4A-deficient mice were born in an expected normal Mendelian distribution, were viable and had normal survival. This was also compatible with the grossly normal appearance of the C4.4A^−/−^ mice and the absence of obvious overt alterations of the various squamous epithelia of the body in young as well as aging mice (Figs 4 and 5). Such a modest functional impact could be the consequence of redundancy of proteins with similar functions already present in the squamous epithelia or upregulated upon C4.4A deficiency. The relevance of this scenario is documented in several independent mouse studies with gene ablation of *e.g.* individual cytokeratins of the epidermis, showing that deletion of CK14 or CK10 are partially compensated for by CK15 and CK14, respectively^31^,^32^. Our mRNA expression profiling on homeostatic skin did, however, not provide any obvious candidates for such functional redundancy (Supplementary tables S2 & S3). Sequencing extracts from whole mouse ears were chosen to obtain information on the impact of C4.4A ablation on homeostatic skin representing both epidermis as well as the underlying connective tissue. Only few genes were found dysregulated by the C4.4A deficiency *per se* and all presented rather modest amplitudes, which is congruent with the absence of an overt, manifest pathology in the affected squamous epithelia.

Both male and female C4.4A^−/−^ mice were slightly lighter than littermate C4.4A^+/+^ controls, and the weight difference of app. 6% was sustained over time in young and adult mice. In the aging female mice, the weight difference progressively increased, and by one year of age, the weight difference could largely be ascribed to differences in both abdominal and subcutaneous fat mass (Fig. 3). This could indicate a direct or indirect role for C4.4A in energy metabolism. Weight phenotypes are observed in a large number of gene-deficient mice^33^, but the actual amount of genes being directly involved and responsible for energy metabolism is uncertain. To determine if differences in metabolic parameters or food consumption were directly correlated to the observed leanness of C4.4A^−/−^ mice we compared their energy expenditure by indirect calorimetry assessment using metabolic cages. The most pronounced difference we observed was a 13.8% reduction in the overall food intake of female C4.4A^−/−^ mice (p < 0.0001), which is in accordance with their smaller body weights. A similar difference was however not observed for male mice. Differences between genotypes in locomotion activity, respiratory quotient or energy expenditure (Supplementary Fig. S3) were either non-significant or considered too subtle to be of functional importance. Consistent with this we found no differences in mRNA levels for genes involved in adipocyte differentiation (*C/ebpβ* and *Ppar γ*) or adaptive thermogenesis (*Ucp1*) in neither white nor brown adipose tissue (Supplementary Fig. 4S).

Pertaining to defects in energy metabolism associated to proteins predominantly expressed in the epidermis, it is worth mentioning that a mouse line deficient in the soluble single LU domain protein SLURP1 presents a pronounced metabolic phenotype^6^. The molecular mechanism underlying these observations, however, still remains unclear. In the present case, one could envisage that the C4.4A^−/−^ mice had a modest functional impairment of the squamous epithelia integrity, undetected by our histological and subcellular examination. No differences between the genotypes, however, were observed when measuring TEWL in newborn pups (Supplementary Fig. S2), suggesting that either the barrier efficiency of the skin is intact or that the defect is too modest to be detected by this assay. In addition to its role in maintenance of fluid homeostasis, the epidermis also provides a first line defence against infectious assaults. As our global mRNA sequencing revealed a tendency towards modest upregulation of transcripts primarily expressed by inflammatory cells in the C4.4A^−/−^ mice, the epidermal barrier integrity could nonetheless be moderately compromised in this respect.

It has become generally accepted that the widely used gene disruption strategy in mice, which involves the partial or complete deletion of the target gene together with concomitant insertion of a drug selection cassette, can cause disturbances in the regulation of adjacent genomic areas. Such manipulations can disrupt or intensify expression of genes located near the target gene and therefore add a confounding factor to the interpretations of the observed phenotypes^34^. Strikingly, we found that the five closest neighbouring genes located within a range of app. 80 kb of *Lypd3* (*Ethe1*, *Tex101*, *Phldb3*, *BC049730*, *LOC102640044*) were significantly dysregulated in the C4.4A^−/−^ mice (Supplementary tables S2 & S3). Downregulation of the mitochondrial dioxygenase Ethe1 was of particular concern in this study, as Ethe1-deficient mice have a severe phenotype, showing postnatal growth arrest and early death as a consequence of toxic levels of sulphide accumulation^26^. Even though the weight phenotype of the C4.4A^−/−^ mice was mild, it could be a consequence of sub-lethal elevations in the sulphide levels due to a borderline rate limitation of the Ethe1 catalytic activity enforced by its attenuated protein synthesis. Importantly, steady-state levels of thiosulfate in serum and urine were within the normal range for all genotypes, eliminating the activity of Ethe1 as a driving causative factor for the phenotypes in the C4.4A^−/−^ mice.

This newly generated C4.4A-deficient mouse line provides an optimal platform for a thorough examination of the suggested role of C4.4A in tumour invasion and progression. Wound healing is often used as a surrogate model of cancer invasion, since a number of processes are shared such as extracellular matrix degradation, proliferation and migration^35^. Examining the *in vivo* healing of incisional back skin wounds did not reveal an effect of C4.4A deficiency in female mice, but a significantly delayed wound healing was nevertheless observed in the male C4.4A^−/−^ mice (Fig. 6). Gender-specific differences in wound healing have previously been seen in other gene-deficient mice such as the plasminogen-deficient mouse line^36^. Male skin is inherently different from female skin as it has an obvious thicker dermis, marked thinner hypodermis and slightly thinner epidermis. These differences arise from specific effects of the sex steroids oestrogen and testosterone^37^. Testosterone furthermore delays wound healing, which is accompanied by an increased inflammatory response^38^. It is accordingly tempting to speculate that increased inflammation, in addition to C4.4A deficiency, could have an additive gender-specific effect in delaying the wound healing process in male mice.

Interestingly, C4.4A deficiency seemed to affect the incidence of invasive bladder carcinomas, since fewer C4.4A^−/−^ mice (44%) developed invasive carcinomas compared to C4.4A^+/+^ littermates (83%). This was, however, not recapitulated by a genotype-dependent difference on neither the incidence of early hyperplastic lesions (Fig. 7) nor in the size of tumour lesions (Fig. 8), which could indicate that C4.4A is important for the invasive process, but not the malignant potential *per se*. This hypothesis fits with the difference being found primarily in the occurrence of early invasive lesions, *i.e.* invasive into the connective tissue (C4.4A^−/−^; 13% and C4.4A^+/+^; 39%) and not later, more malignant lesions, *i.e.* invasive into the muscle layer (C4.4A^−/−^; 31% and C4.4A^+/+^; 44%). Along the same line of evidence, C4.4A is highly expressed in the invasive front of both benign keratoacanthomas and malignant SCCs of the human skin, suggesting a role for C4.4A in the invasive process independent of malignancy^10^. It should, nevertheless, be emphasized that this hypothesis needs further experimental verification. A pronounced impact from stromal expression of C4.4A on tumour take or growth was not obvious from subcutaneous engraftment studies with Lewis Lung carcinoma cells (Supplementary Fig. S7).

In conclusion, our data reveal that C4.4A is dispensable for normal squamous epithelial development and differentiation. Still, our challenge studies show that C4.4A deficiency delays skin wound healing kinetics in males and indicate that female C4.4A-deficient mice tend to develop fewer invasive bladder carcinomas compared to the corresponding littermate controls. One important question that still awaits experimental validation is whether the strong prognostic impact of C4.4A expression on the survival of cancer patients with *e.g.* lung adenocarcinomas reports on a *bona fide* functional causal relationship. For this purpose, several mouse models exist for induction of non-small cell lung cancer of the adenocarcinoma subtype both by genetic aberrations and by chemical insult^39^,^40^ which could be combined with the presently C4.4A-deficient mouse line. The absence of profound overt phenotypes in both juvenile and aging C4.4A-deficient mice is considered beneficial for the future use of these animals in studies aiming at elucidating the possible impact of C4.4A-expression on the progression of lung adenocarcinomas whether these lesions are induced genetically or by external exposure to carcinogens. These mice may also serve as excellent controls in preclinical trials for specificity and target-unrelated toxicity of a given intervention strategy targeting C4.4A. The use of C4.4A as a pharmaceutical target is currently being explored in a phase-1 clinical trial (NCT02134197) where patients with advanced solid tumours are treated with a monoclonal anti-C4.4A antibody conjugated to the antimitotic drug auristatin E (BAY1129980).

## Methods

### Antibodies

Rabbit polyclonal antibodies (pAb) against human C4.4A and Haldisin were produced and purified in house as previously described^23^,^41^. Rabbit monoclonal antibody (mAb) against Ki67 (ab16667) as well as rabbit pAb towards pan-cytokeratin (CK) (ab9377) and against loricrin (ab24722) were purchased from Abcam (Cambridge, UK). Rabbit pAb anti-CK10 (PRB-159P) and anti-CK14 (PRB-155P) were from Covance (Princeton, NJ). Horseradish peroxidase-labelled EnVision rabbit reagent (K4003) was purchased from Dako (Carpinteria, CA). Goat anti-rabbit pAb (A10533) used as secondary antibody for electron microscopy was from Invitrogen (Eugene, OR).

### Generation of C4.4A-deficient mice, genotyping and breeding

*Lypd3*-targeted ES cells (Lypd3^tm1(KOMP)Vlcg^) were generated by Regeneron Pharmaceuticals, Inc. for the NIH-funded Knockout Mouse Project (KOMP) and obtained from the NCRR-NIH-supported KOMP Repository (www.komp.org)^42^.

The parental ES cell line, VGB6 (formerly B6A6), was isolated from the C57BL/6NTac mouse strain. A 4068 bp fragment covering position 25,421,612–25,425,679 (exons 1–5, NCBI mouse genome build 37) of *Lypd3* on chromosome 7 (whole gene position 25,421,589–25,426,137) was replaced by a β-galactosidase-neomycin cassette using a targeting vector and homologous recombination (Fig. 1A). The detailed information on the knock-out strategy used can be found on www.komp.org with the project ID VG13176. Targeted ES cells were injected into the cavity of Balb/c blastocysts, which were subsequently transferred into pseudopregnant NMRI female mice. Chimeric male offspring was mated to C57BL/6J females to generate heterozygous progeny. These mice were subsequently interbred to generate C4.4A^−/−^, C4.4A^+/−^ and C4.4A^+/+^ littermates. Genotyping of mice was performed by standard PCR amplification of DNA from tail tip biopsies using the HotStarTaq Master Mix (Qiagen, Hilden, Germany) and 0.5 μM of each of the primers: mLypd3 exon5 5′ (5-CAACGTGACCGTGTCCTTACCTG-3) and mLypd3 exon5 3′ (5-CTGGGCCTCTCTCAGCCAGTAG-3), to detect the wild-type *Lypd3* allele (806 bp), and primers SU (5-ACCCTAGCAGACCCTGAATG-3) and LacZRev (5-GTCTGTCCTAGCTTCCTCACTG-3), to detect the targeted *Lypd3* allele (464 bp), see Fig. 1A. The PCR fragments were visualized with UV light on a 2% agarose gel containing 0.5 μg/ml ethidium bromide as shown in Fig. 1B.

### Animal housing and ethics statement

All mice were housed in IVC cages in a certified facility at the University of Copenhagen, Denmark, with 12-hour light/dark cycle and *ad libitum* standard chow diet/water access. Experimental animals were monitored daily by trained animal caretakers and were euthanized by cervical dislocation upon signs of distress. All experiments were performed according to institutional and national guidelines and approved by the Danish Animal Experiments Inspectorate (license numbers 2012-15-2934-00062 and 2013-15-2934-00851).

### Observation and weighing of mice

Two cohorts of male and female littermate-controlled mice were bred using C4.4A^+/−^ mice, on a mixed C57BL/6N/C57BL/6J background, as breeding pairs. Cohort I consisted of F2 female C4.4A^−/−^ (n = 14), C4.4A^+/−^ (n = 15) and C4.4A^+/+^ (n = 16) mice as well as male C4.4A^−/−^ (n = 11), C4.4A^+/−^ (n = 14) and C4.4A^+/+^ (n = 14) mice bred from C4.4A^+/−^ F1 mice. This cohort was followed for 26 weeks, *i.e.* from weeks 5 to 30 of age. During this time, the mice were observed macroscopically for overt phenotypic changes, and the weight of the mice was recorded once a week. At 30 weeks of age, all male mice as well as female C4.4A^+/−^ mice were sacrificed and tissue was isolated. Female C4.4A^−/−^ and C4.4A^+/+^ mice were followed for another 22 weeks, *i.e.* until 52 weeks of age, and weighed every second week. At the end of the observation period, a CT scanning as well as a whole body composition analysis were performed on 10 female mice of each genotype. The CT data were obtained using a MicroCAT II tomograph (Siemens Medical Solutions, Malvern, PA), and the whole-body composition analysis was performed using a quantitative magnetic resonance whole-body composition analyser (EchoMRI, Echo Medical Systems, Houston, TX). For CT scanning, the radiographic tube with a 0.5 mm aluminium filter was set at 40 kVp, a tube current of 500 mA, and an exposure time of 700 ms per projection. The pixel size was 0.095 × 0.095 × 0.095 mm. Bone length was measured from the CT scan using the Inveon visualization software (Siemens Medical Solutions) and bone mineral densities were assessed from the CT data in Hounsfield units^43^.

Cohort II consisted of F3 female C4.4A^−/−^ (n = 12), C4.4A^+/−^ (n = 31) and C4.4A^+/+^ (n = 10) mice as well as male C4.4A^−/−^ (n = 11), C4.4A^+/−^ (n = 33) and C4.4A^+/+^ (n = 20) mice bred from C4.4A^+/−^ F2 breeding pairs. The F3 mice were monitored from birth (day 0) until 6 weeks of age. The mice were observed for macroscopically overt phenotypes and weighed every seventh day calculated from their day of birth.

### Tissue preparation

At the end of a given experimental period or in cases of premature euthanasia due to ethical reasons, the mice were anesthetized by intraperitoneal administration of 0.1 ml/10 g mouse of a 1:1 mixture of Hypnorm (Fluanison 5 mg/ml and Fentanyl 0.1 mg/ml) and Dormicum (Midazolam 5 mg/ml). The animals were perfused by intracardial injection of 10 ml cold phosphate-buffered saline (PBS), followed by 10 ml cold 4% formaldehyde in buffered PBS before isolation of tissue. Resected tissue was fixed in 4% formaldehyde in buffered PBS for 24 hours before standard paraffin embedding.

### H&E and immunoperoxidase staining

H&E and immunoperoxidase staining was made on 3.5 μm thick paraffin-embedded tissue sections which were de-paraffinized in xylene and hydrated through ethanol/water solutions before further treatment. H&E staining was performed by staining in Mayer’s haematoxylin (5 min), washing in tap water (5 min), staining with eosin (5 min) and rinsing in tap water (1 min).

Sections for immunoperoxidase staining were either incubated with 5 μg/ml proteinase K for 10 min at 37 °C for antigen retrieval of C4.4A, Haldisin, CK14, panCK and loricrin or pre-treated at 98 °C for 10 min in 10 mM sodium citrate, pH 6.0, for antigen retrieval of CK10 and Ki67. Endogenous peroxidase activity was blocked by incubation in 1% (v/v) hydrogen peroxide for 15 min. The sections were washed in Tris-buffered saline (TBS) (50 mM Tris, 150 mM NaCl, pH 7.6) containing 0.5% (v/v) Triton X-100 before overnight incubation at 4 °C with the primary antibodies diluted in Antibody Diluent (S0809, Dako, Glostrup, Denmark) to the following concentrations; pAb C4.4A: 2.5 μg/ml; pAb Haldisin: 1 μg/ml; pAb CK10: 2.2 μg/ml; pAb CK14: 0.1 μg/ml; pAb loricrin: 0.3 μg/ml; mAb Ki67: 1:100; panCK: 1:650. Subsequently, the primary antibodies were detected with EnVision rabbit reagent, and the sections were developed with NovaRed (SK-4800, Vector Laboratories, Burlingame, CA) for 9 min as specified by the manufacturer. The sections were then counterstained with Mayer’s haematoxylin for 30 sec. Both H&E and immunoperoxidase stained sections were finally dehydrated in ethanol solutions before mounting in pertex.

### Immunogold electron microscopy on ultracryosections

6 weeks old female C4.4A^−/−^ and C4.4A^+/+^ mice were perfused in PBS and 4% formaldehyde in buffered PBS as described above. Tissues were cut into 1 mm^2^ pieces and fixed for 2 hours at 4 °C before being immersed in 2.3 M sucrose in 0.1 M sodium phosphate buffer, pH 7.2 overnight, and mounted on a metal pin using liquid nitrogen. Ultracryosections of app. 90 nm were cut using an RMC 6000XL ultracryomicrotome and placed on formvar-coated nickel grids. The cryosections were treated in TBS (pH 7.4) with 0.02 M glycin and subsequently in dilution and wash (D&W) buffer (0.05 M Tris, pH 7.4, containing 0.15 M NaCl, 0.5% (w/v) ovalbumin, 0.1% (w/v) gelatin, 0.05% (v/v) Tween-20 and 0.2% (w/v) Teleostean gelatin), before they were incubated with 2 μg/ml rabbit pAb C4.4A overnight at 4 °C in a humid chamber. Subsequently, the sections were washed in D&W buffer prior to 30 min incubation with 13 nm gold particle-coupled goat anti-rabbit pAb (A10533, Invitrogen, Eugene, OR) at room temperature. The gold particles were prepared and conjugated to antibodies as previously described^44^,^45^. The ultracryosections were extensively washed in D&W buffer followed by TBS and finally water before being stained 3 times 30 sec in 0.4% (v/v) uranyl acetate in 2% methylcellulose. The sections were examined using a Zeiss EM 900 transmission electron microscope equipped with a Zeiss Mega View II camera system.

### Electron microscopy on Epon-embedded tissue

6 weeks old female and 10 weeks old male C4.4A^−/−^ and C4.4A^+/+^ mice (1 of each) were perfused with 10 ml PBS followed by 10 ml Karnovsky fixative (2% paraformaldehyde and 3% glutaraldehyde in 0.1 M sodium phosphate buffer, pH 7.2). Tissues cut into 1 mm^2^ pieces were fixed in Karnovsky fixative for 2 hours at 4 °C and further treated 1 hour in 1% (w/v) osmium tetraoxide in 0.1 M sodium phosphate buffer, pH 7.2, dehydrated in a series of ethanol solutions and finally 100% acetone. Epon TAAB 812 embedding resin (WC/DK4096, Th-Geyer, Roskilde, Denmark) was prepared according to the manufacturer’s instructions. The tissues were incubated in a 1:1 Epon:acetone solution overnight at room temperature and embedded in Epon, which was hardened at 60 °C for 3 days. Ultrathin sections of app. 50 nm were cut in an LKB Ultrotome III and collected on nickel grids. The sections were finally stained with 1% (v/v) uranyl acetate in water for 5 min and lead citrate^46^ for 45 sec before being examined with the equipment described above.

### Wound healing

Cohorts of 6–8 weeks old female C4.4A^−/−^ (n = 11) and C4.4A^+/+^ (n=13) as well as male C4.4A^−/−^ (n = 12) and C4.4A^+/+^ (n = 13) littermates were anesthetized and standardized 20 mm long full-thickness incisional skin wounds were made mid-dorsally with a scalpel. To ensure uniform wounds, the wounding was carried out by a single person. The wounds were visually examined and the wound length measured by a calliper every second day until closure. Healing was defined as a complete loss of the wound scab and closure of the incision edges with reestablishment of the covering epidermis. The length was defined the longest distance covered by the scab. The mice were caged individually until sacrifice at the time of wound closure. Wounds were furthermore isolated for histological analysis after PBS and formalin perfusion of the mice as described above.

### Treatment with N-butyl-N-(4-hydroxybutyl)nitrosamine (BBN)

Female C4.4A^−/−^ and C4.4A^+/+^ littermates (8–12 weeks old) received either 0.05% (v/v) BBN (B0938, TCI Europe N.V., Zwijndrecht, Belgium) in their drinking water or drinking water without BBN (control mice). Precursor lesions (hyperplasia) were induced by 4 weeks BBN exposure (C4.4A^−/−^ (n = 8) and C4.4A^+/+^ (n = 9)), whereas more severe lesions, including carcinomas, were induced by a 26 weeks experiment (C4.4A^−/−^ (n = 16) and C4.4A^+/+^ (n=18)) of 14 weeks of BBN exposure followed by 12 weeks of water^29^. C4.4A^−/−^ (n=6) and C4.4A^+/+^ (n=3) control mice received normal drinking water for 26 weeks. Water bottles were changed twice a week. Before sampling of the bladders, the mice were anesthetized and the bladders distended by *in situ* fixation using 100 μl 4% formaldehyde in buffered PBS injected via a 24G catheter. The urethral orifice was closed by a thread, and the ballooned bladders were fixed for 24 hours, before being cut into halves and embedded in paraffin. Morphologic alterations of the bladders were evaluated on H&E- as well as panCK-stained sections by an experienced pathologist (ODL) according to the following parameters: histological subtype and non-invasiveness, invasiveness into connective tissue or into muscle layer. For ethical reasons, 3 C4.4A^−/−^ and 3 C4.4A^+/+^ mice were euthanized prematurely: 2 C4.4A^−/−^ mice (8 and 73 days before schedule) and 2 C4.4A^+/+^ mice (19 and 24 days before schedule) were sacrificed due to the tumour burden. One C4.4A^−/−^ mouse (10 days before schedule) and one C4.4A^+/+^ mouse (15 days before schedule) were sacrificed due to tumour burdens obstructing the ureter and urethra, respectively.

### Computer-assisted morphometric analysis

Image analysis was performed in a blinded set-up using the Visiomorph software package (Visiopharm, Hørsholm, Denmark). Proliferation of the bladder urothelium was quantified by counting Ki67-positive and total nuclei on Ki67-stained tissue sections. Bladder tumour areas were quantified on H&E-stained sections of both paraffin-embedded bladder halves and the mean tumour area was calculated.

### Statistical analysis

The hypothesis testing for a Mendelian distribution of genotypes was done using the *χ*^2^-test. The analysis of experiments with repeated measurements on the same subjects (weight, wound healing, TEWL, tumour volume, food intake, respiratory quotient, locomotion activity, energy expenditure) were done using linear mixed modelling in order to account for the correlated measurements. All weights and wound lengths were log transformed for analysis in order to have normally distributed data and back-transformed for presentation. In addition, the best fits were, in some cases, obtained with the time of measurement log transformed. The variances of weight on the log scale (natural) were estimated using linear models, and the CV was therefore: CV=SQRT(exp(variance)-1). This CV is often designated as the geometric CV. The fat and lean mass experiments were performed using linear mixed modelling with the dependent variable (fat mass (g), lean mass (g), fat mass (%), lean mass (%)) on the original scale. The fraction of Ki67-positive nuclei, thiosulfate concentration, femur length, femur density and qPCR data were compared between genotypes using t-tests. The bladder tumour area was analysed with the *χ*^2^-test. All estimates are presented on the original scale with standard deviations or standard errors of the mean where applicable. The level of significance was 5%. All calculations were done using SAS (v9.3, SAS Institute, Cary, N.C., USA).

## Additional Information

**How to cite this article**: Kriegbaum, M. C. *et al.* C4.4A gene ablation is compatible with normal epidermal development and causes modest overt phenotypes. *Sci. Rep.* **6**, 25833; doi: 10.1038/srep25833 (2016).

## Supplementary Material

Supplementary Information
