# Supplementary material for: C4.4A gene ablation is compatible with normal epidermal development and causes modest overt phenotypes
Source: Sci Rep. 2016 May 12;6:25833. doi: 10.1038/srep25833 (PMC4864438; doi:10.1038/srep25833)
Supplement: Supplementary Information [file srep25833-s1.doc]

**C4.4A gene ablation is compatible with normal epidermal development and cause modest overt phenotypes**

**Mette Camilla Kriegbaum^1,2^, Benedikte Jacobsen^1,2^, Annette Füchtbauer^3^, Gert Helge Hansen^4^, Ib Jarle Christensen^1,2^, Carsten Friis Rundsten^2^, Morten Persson^5,10^, Lars Henning Engelholm^1,2^, Andreas Nygaard Madsen^6^, Ivano Di Meo^7^, Ida Katrine Lund^1,2^, Birgitte Holst^6^, Andreas Kjaer^5,10^, Ole Didrik Lærum^8,9^, Ernst-Martin Füchtbauer^3,10^, and *Michael Ploug^1,2,10^**

^1^The Finsen Laboratory, Rigshospitalet, Copenhagen, Denmark

^2^Biotech Research and Innovation Centre (BRIC), University of Copenhagen, Copenhagen, Denmark

^3^Department of Molecular Biology and Genetics, Aarhus University, Aarhus, Denmark

^4^Department of Cellular and Molecular Medicine, University of Copenhagen, Copenhagen, Denmark

^5^Department of Clinical Physiology, Nuclear Medicine & PET and Cluster for Molecular Imaging, Rigshospitalet and University of Copenhagen, Copenhagen, Denmark

^6^Deparment of Pharmacology, University of Copenhagen, Denmark

^7^Division of Molecular Neurogenetics, IRCCS Foundation Neurological Institute “Carlo Besta”, Milano, Italy

^8^Department of Pathology, Haukeland University Hospital, Bergen, Norway

^9^Department of Clinical Medicine, The Gade Laboratory of Pathology, University of Bergen, Norway

^10^The Danish National Research Foundation (Danish-Chinese Centre for Proteases and Cancer)

Corresponding author*

Michael Ploug, The Finsen Laboratory, Rigshospitalet section 3735, Copenhagen Biocenter, Ole Maaløes Vej 5, Room 3.3.31, DK-2200 Copenhagen N, Denmark; Phone: (45) 35456037; Fax: (45) 35453797;

E-mail: m-ploug@finsenlab.dk

**Supplementary information**

**Supplementary methods**

**Thiosulfate measurements**

Thiosulfate concentrations were measured in serum and urine collected from C4.4A^-/-^ (n=8) and C4.4A^+/+^ (n=8) mice essentially as described^1^. Serum was prepared from heart blood drawn from anesthetized mice. The blood was left to coagulate at room temperature for 1 hour, centrifuged at 3000 x g for 15 min, and the serum was subsequently collected. The presence of thiosulfate was detected by addition of 2.9 mM monobromobimane to both urine and serum samples. The fluorescent products were separated by HPLC with a gradient pump and an increasing gradient of methanol, using a LiChrospher 60 RP-select B, 125-4 (5 μm) column (1508290001, MERCK, Darmstadt, Germany). Fluorescence emission at 480 nm was detected online after excitation at 380 nm.

**Indirect calorimetry measurements**

Cohorts of 12-14 weeks old C4.4A^-/-^ mice (males n=5 and females n=9) and littermate control C4.4A^+/+^ (males n=6 and females n=8) mice were individually housed in habituation cages for more than 7 days. All mice were transferred to a 16-chamber indirect calorimetry system (PhenoMaster; TSE Systems, Bad Hamburg, Germany) and respiratory exchange ratio (RER), total locomotion activity (beam breaks), energy expenditure, and food intake were simultaneously measured for each mouse. Data for 3 nights and 2 day phases are shown.

**Resection of adipose tissue and isolation of mRNA**

After metabolic assessment all mice were allowed to acclimatise for 1 week before retroperitoneal and gonadal white adipose tissue and brown adipose tissue were resected, weighed, snap frozen in liquid N_2_, and stored at –80°C until use. Total RNA was isolated from each sample using TRIzol® reagent (ThermoFisher Scientific, Copenhagen, Denmark). Briefly each tissue sample was crushed using a dry-ice cooled ceramic mortar; frozen tissue powder was immediately transferred to 1 ml TRIZol reagent for each 100 mg tissue. Tissue was further disrupted using a polytron homogenizer and subsequent steps in RNA isolation was done according to manufacturer’s description. cDNA was synthesized by ProtoScripts II First Strand cDNA Synthesis Kit (New England BioLabs, Ipswich, UK) using standard conditions and Oligo d(T)_23_ VN as primer.

**Quantitative real-time PCR of selected adipocyte genes**

Relative mRNA levels were measured by quantitative PCR (qPCR) with a Roche Lightcycler 480, using SYBR Green (Roche, Hvidovre, Denmark). 3 primer sets for each target gene were validated with respect to amplification efficiency and primer dimer formations, and primers with >95% efficiency and single amplification products (as analysed by melt curve genotyping) were used in subsequent analysis (primers listed in Supplementary table S4). The relative levels of gene expression from different samples were compared by the ∆∆C_t_ method using β_2_-microglobulin (β_2_m) and Actin gamma 1 (Actγ1) as reference genes and triplicate amplifications for all primer pairs.

Neither *Lypd3* nor *Lypd5* were amplified from adipose tissue generated cDNA, indicating that C4.4A and Haldisin are not expressed in adipose tissue. Semi-quantitative PCR on mRNA isolated from skin and esophagus were used as positive controls. RNA and cDNA were isolated and generated as described above. PCR was done using the listed primers, Hot Star Taq Plus Master Mix Kit (Qiagen, Copenhagen, Denmark) and a GeneAmp PCR System 9700 (Applied Biosystem) using the following temperature profile; 15 min at 95°C, 36 cycles with 45 sec. at 94°C, 45 sec. at 60°C and 45 sec. at 72°C followed by a final 10 min. elongation at 72°C. PCR products were then analysed on a 2% agarose gel containing ethidium bromide.

**Skin barrier assay**

To measure TEWL, newborn C4.4A^-/-^ (n=12), C4.4A^+/-^ (n=12) and C4.4A^+/+^ (n=17) littermate pups of maximum 5 hours of age, generated by heterozygous breeding, were incubated at 37°C for 6 hours and weighed every hour for 6 hours. In case of urination, the pups were excluded from the experiment. TEWL was determined as the calculated percentage weight loss over time.

**Global mRNA sequencing**

8 weeks old C4.4A^-/-^ and C4.4A^+/+^ littermate female and male mice (3 of each) were anesthetized, and the ears were removed and immediately snap-frozen in liquid nitrogen. The tissue was homogenized and total RNA extracted with TRIzol Reagent (Life technologies, Carlsbad, CA) according to the manufacturer’s instructions. The samples were treated with DNase before the RNA quality was determined by the RNA integrity number using the Agilent 2100 Bioanalyzer and RNA pico Chips from Agilent Technologies (5067-1513, Waldbronn, Germany). The TruSeq RNA Sample Preparation kit v2 from Illumina (RS-122-2001, San Diego, CA) was used, and the TruSeq Sample Preparation v2 Guide, Low Sample Protocol was followed to generate cDNA libraries for sequencing. In brief, mRNA was purified from 4 ng total RNA using poly-T oligo-attached Agencourt AMPure XP magnetic beads (A63881, Beckman Coulter, Brea, CA) and fragmented to 350-500 bp fragments. cDNA libraries were synthesized, and indexing adapters were ligated to the 3’-end. The adapter-ligated cDNA libraries were run on a 2% agarose gel according to Illumina’s “Purify Ligation Products (gel method only)” protocol, and ligation products of 350-500 bp were cut from the gel and purified with the QIAquick Gel Extraction Kit (28704, Qiagen, Hilden, Germany) as specified by the manufacturer. The selected and purified libraries were amplified by PCR, and the final concentrations were determined using a Qubit fluorometer (Invitrogen, Eugene, OR). The base pair sizes of the cDNA libraries were confirmed using the Agilent 2100 Bioanalyzer and DNA Chips from Agilent Technologies (5067-1504, Waldbronn, Germany). The 6 male- and 6 female-derived libraries, respectively, were pooled and sequenced on two separate lanes at a HiSeq2000 from Illumina (San Diego, CA) using paired-end sequencing. All handling was done under DNase- and RNase-free conditions.

**mRNA sequencing analysis**

The RNAseq data were aligned to the UCSC mm10 reference genome using the STAR aligner^2^ with default parameters except for --outFilterMismatchNoverLmax 0.04 and --outFilterMatchNmin 16. The htseq-counts program from the HTseq python package^3^ was used to calculate the observed counts for each gene, which were then analysed in R using the DESeq package library (version 1.16.0)^4^. The nbinomTest function in DESeq was used to test each of the pairwise comparisons: C4.4A^+/+^ versus C4.4A^-/-^ in males and females, respectively. Principal component analysis was also performed using the tools in the DESeq library; in particular, plots were drawn using the plotPCA function with default parameters. Of note is that default parameters for plotPCA base the illustration on the data from the 500 features with the highest variance.

**Growth of transplanted lung carcinoma cells on mice**

8-9 weeks old female C4.4A^+/+^ and C4.4A^-/-^ littermate mice (5 of each genotype) were subcutaneously injected with 2.5x10^6^ Lewis lung carcinoma (LLC) cells on the right flank at day 0. The tumour cells were cultured in Dulbecco’s modified Eagle’s medium (DMEM) as previously described^5^. Tumour growth was followed and measured using a calliper by an investigator unaware of the mouse genotype. Tumour volume was calculated as 0.5 * length * width * depth.

**Supplementary tables**

Supplementary table S1: Distribution of genotypes

|  | **Males** | | **Females** | |
| --- | --- | --- | --- | --- |
|  | Obs. | Exp. | Obs. | Exp. |
| **C4.4A^+/+^** | 67  (28.5) | 58.75  (25.0) | 42  (25.6) | 41  (25.0) |
| **C4.4A^+/-^** | 118  (50.2) | 117.5  (50.0) | 84  (51.2) | 82  (50.0) |
| **C4.4A^-/-^** | 50  (21.3) | 58.75  (25.0) | 38  (23.2) | 41  (25.0) |
| **Total** | 235 | | 164 | |
| **χ^2^ test** | p=0.29 | | p=0.86 | |

The table shows the distribution of genotypes of the F2 offspring of C4.4A^+/-^ F1 breeding pairs. The numbers in brackets indicate the calculated incidence percentage.

**Supplementary table S2: Dysregulated genes in the C4.4A-deficie**nt female mice as analysed by mRNA sequencing

| **Gene** | **p-value adjusted** | **Fold change** | **Mean reads C4.4A^-/-^** | **Mean reads C4.4A^+/+^** | **Id (NCBI)** |
| --- | --- | --- | --- | --- | --- |
| **Lypd3** | ≈0 | 548.80↓ | 8.69 | 4771.03 | Ly6/Plaur domain containing 3 [MGI:1919684] |
| **Tex101** | 4.26E-128 | 95.52↑ | 496.28 | 5.20 | testis expressed gene 101 [MGI:1930791] |
| **Ethe1** | 1.78E-08 | 2.57↓ | 56.17 | 144.61 | ethylmalonic encephalopathy 1 [MGI:1913321] |
| **Kpna1** | 5.42E-08 | 1.58↑ | 1209.66 | 766.44 | karyopherin (importin) alpha 1 [MGI:103560] |
| **LOC102640044** | 2.39E-06 | 6.84↓ | 10.27 | 70.24 | ncRNA |
| **Mill1*** | 7.25E-06 | 1.84↑ | 337.93 | 183.81 | MHC I like leukocyte 1 [MGI:2179988] |
| **Gm6189** | 7.25E-06 | 3.08↓ | 35.04 | 108.06 | Gm6189 (pseudogene) [MGI:3643154] |
| **Phldb3** | 1.40E-05 | 1.55↓ | 452.97 | 703.93 | pleckstrin homology-like domain, family B, member 3 [MGI:3642959] |
| **Cxcr6*** | 0.0005 | 2.19↑ | 120.14 | 54.95 | chemokine (C-X-C motif) receptor 6 [MGI:1934582] |
| **Pira2*** | 0.0007 | 3.49↓ | 13.54 | 47.32 | paired-Ig-like receptor A2 [MGI:1195970] |
| **Edn2** | 0.0015 | 2.63↓ | 24.19 | 63.52 | endothelin 2 [MGI:95284] |
| **Sprr1b** | 0.0016 | 2.20↓ | 84.06 | 185.32 | small proline-rich protein 1B [MGI:106659] |
| **BC049730** | 0.0018 | NA | 18.55 | 0 | cDNA sequence BC049730 [MGI:2681843] |
| **Krtap3-2** | 0.0031 | 2.34↓ | 172.01 | 401.76 | keratin associated protein 3-2 [MGI:1913958] |
| **Krt24** | 0.0116 | 1.36↓ | 5449.09 | 7390.83 | keratin 24 [MGI:1922956] |
| **Il12rb2*** | 0.0116 | 1.61↑ | 277.89 | 173.12 | interleukin 12 receptor, beta 2 [MGI:1270861] |
| **Cd163l1*** | 0.0125 | 2.25↑ | 172.37 | 76.71 | CD163 molecule-like 1 [MGI:2443796] |
| **Gm13680** | 0.0325 | 1.27↓ | 3969.53 | 5050.53 | Gm13680 (pseudogene) [MGI:3652285] |
| **Retnla*** | 0.0430 | 1.44↑ | 369.41 | 257.31 | resistin like alpha [MGI:1888504] |

The fold change is calculated as up- (↑) or downregulation (↓) in the C4.4A^-/-^ mice. Proteins involved in inflammatory responses are marked with *. The closest neighbours of *Lypd3* are *Tex101*, *Ethe1*, *LOC102640044*, *Phldb3* and *BC049730*.

**Supplementary table S3:** Dysregulated genes in the C4.4A-deficient male mice as analysed by mRNA sequencing

| **Gene** | **p-value adjusted** | **Fold change** | **Mean reads C4.4A^-/-^** | **Mean reads C4.4A^+/+^** | **Id (NCBI)** |
| --- | --- | --- | --- | --- | --- |
| **Lypd3** | ≈0 | 729.41↓ | 5.91 | 4312.16 | Ly6/Plaur domain containing 3 [MGI:1919684] |
| **Tex101** | 2.89e-93 | 218.15↑ | 527.38 | 2.42 | testis expressed gene 101 [MGI:1930791] |
| **LOC102640044** | 1.59e-20 | 9.57↓ | 8.95 | 85.50 | ncRNA |
| **BC049730** | 2.70e-09 | NA | 22.58 | 0 | cDNA sequence BC049730 [MGI:2681843] |
| **Cyp2b23** | 4.55e-09 | 1.95↓ | 308.30 | 602.64 | cytochrome P450, family 2, subfamily b, polypeptide 23 [MGI:3646735] |
| **Defb14*** | 6.41e-09 | 2.86↑ | 131.32 | 45.89 | defensin beta 14 [MGI:2675345] |
| **Il12rb2*** | 1.01e-08 | 2.09↑ | 306.09 | 146.42 | interleukin 12 receptor, beta 2 [MGI:1270861] |
| **Ethe1** | 2.23e-07 | 2.45↓ | 61.67 | 151.22 | ethylmalonic encephalopathy 1 [MGI:1913321] |
| **Krt1** | 2.23e-07 | 1.61↑ | 10840.90 | 6746.04 | keratin 1 [MGI:96698] |
| **Cyp2e1** | 5.04e-05 | 1.89↓ | 224.02 | 423.23 | cytochrome P450, family 2, subfamily e, polypeptide 1 [MGI:88607] |
| **Krtap3-2** | 5.04e-05 | 1.83↓ | 165.60 | 303.12 | keratin associated protein 3-2 [MGI:1913958] |
| **Atp12a** | 0.0019 | 1.56↑ | 634.07 | 407.43 | ATPase, H+/K+ transporting, nongastric, alpha polypeptide [MGI:1926943] |
| **Galnt15** | 0.0033 | 1.45↓ | 2222.49 | 3228.88 | UDP-N-acetyl-alpha-D-galactosamine: polypeptide N-acetylgalactosaminyl-transferase 15 [MGI:1926004] |
| **Alox8** | 0.0033 | 4.01↑ | 59.04 | 14.71 | arachidonate 8-lipoxygenase [MGI:1098228] |
| **Il1f6*** | 0.0041 | 1.71↑ | 224.78 | 131.18 | interleukin 1 family, member 6 [MGI:1859324] |
| **Dsg1c** | 0.0152 | 2.10↓ | 39.20 | 82.16 | desmoglein 1 gamma [MGI:2664358] |
| **Il18*** | 0.0280 | 1.42↑ | 1137.10 | 801.61 | interleukin 18 [MGI:107936] |
| **2310042E22Rik** | 0.0350 | 1.72↑ | 153.62 | 89.37 | RIKEN cDNA 2310042E22 [MGI:1913811] |
| **Mucl1** | 0.0350 | 1.90↑ | 100.56 | 52.84 | mucin-like 1 [MGI:98393] |
| **Cdk5rap1** | 0.0350 | 1.66↑ | 224.72 | 135.38 | CDK5 regulatory subunit associated protein 1 [MGI:1914221] |
| **Notum** | 0.0394 | 1.52↓ | 202.75 | 308.18 | notum pectinacetylesterase homolog (Drosophila) [MGI:1924833] |
| **Cd163l1*** | 0.0495 | 1.82↑ | 115.40 | 63.49 | CD163 molecule-like 1 [MGI:2443796] |
| **Serpina3j*** | 0.0495 | 1.67↓ | 244.56 | 408.26 | serine (or cysteine) peptidase inhibitor, clade A (alpha-1 antiproteinase, antitrypsin), member 3J [MGI:2182843] |

The fold change is calculated as up- (↑) or downregulation (↓) in the C4.4A^-/-^ mice. Proteins involved in inflammatory responses are marked with *. The closest neighbours of *Lypd3* are *Tex101*, *LOC102640044*, *BC049730* and *Ethe1*.

Supplementary table S4: List of primers used for qPCR analysis

| **Gene** | **Alias** | **Primer name** | **Sequence** |
| --- | --- | --- | --- |
| **β_2_-microglobulin** | β_2_m | β_2_m -for | CTGACCGGCCTGTATGCTAT |
|  |  | β_2_m -rev | CAGTCTCAGTGGGGGTGAAT |
| **Actin gamma 1** | *Actγ1* | *γ-Act*-for | CTCTTCCAGCCTTCCTTCCT |
|  |  | *γ-Act* -rev | TGCTAGGGCTGTGATCTCCT |
| **Uncoupling Protein 1** | *Ucp1* | *Ucp1* 1 F | AAATACTGGCAGATGACGTCC |
|  |  | *Ucp1*_1 R | CTTGGTACGCTTGGGTACTG |
|  |  | *Ucp1*_2 F | CTTCTCAGCCGGAGTTTCAG |
|  |  | *Ucp1*_2 R | CTTCACCTTGGATCTGAAGGC |
|  |  | *Ucp1*_3 F | CACTTTGGAAAGGGACGACC |
|  |  | *Ucp1*_3 R | TGAGGTCATATGTTACCAGCTC |
| **Peroxisome proliferator-activated receptor gamma** | *Pparγ* | *Pparγ* _F1 | GATCTTAACTGCCGGATCCA |
|  |  | *Pparγ* _R1 | ACCTGATGGCATTGTGAGAC |
| **CCAAT/enhancer-binding protein beta** | *C/ebpβ* | C/ebpβ _F1 | AAGATGCGCAACCTGGAG |
|  |  | C/ebpβ _R1 | GCTGCTCCACCTTCTTCTG |
| **Ly6/PLAUR domain-containing protein 3** | *Lypd3* | *Lypd3*_1 F | CTGGCCTTCTTTCAGCTACAG |
|  |  | *Lypd3*_1 R | TCTCATTGCCTGCAGGGT |
|  |  | *Lypd3*_3 F | GGATGAAGACAGTCAAATGTGGT |
|  |  | *Lypd3*_3 R | AATTGCCCGTGGATGGTC |
| **LY6/PLAUR domain containing 5** | *Lypd5* | *Lypd5*_F1 | CCACCCTGAAGACTGTTCC |
|  |  | *Lypd5*_R1 | GAGAAGTTGCCAATGTTCATCC |

**Supplementary figures**

**
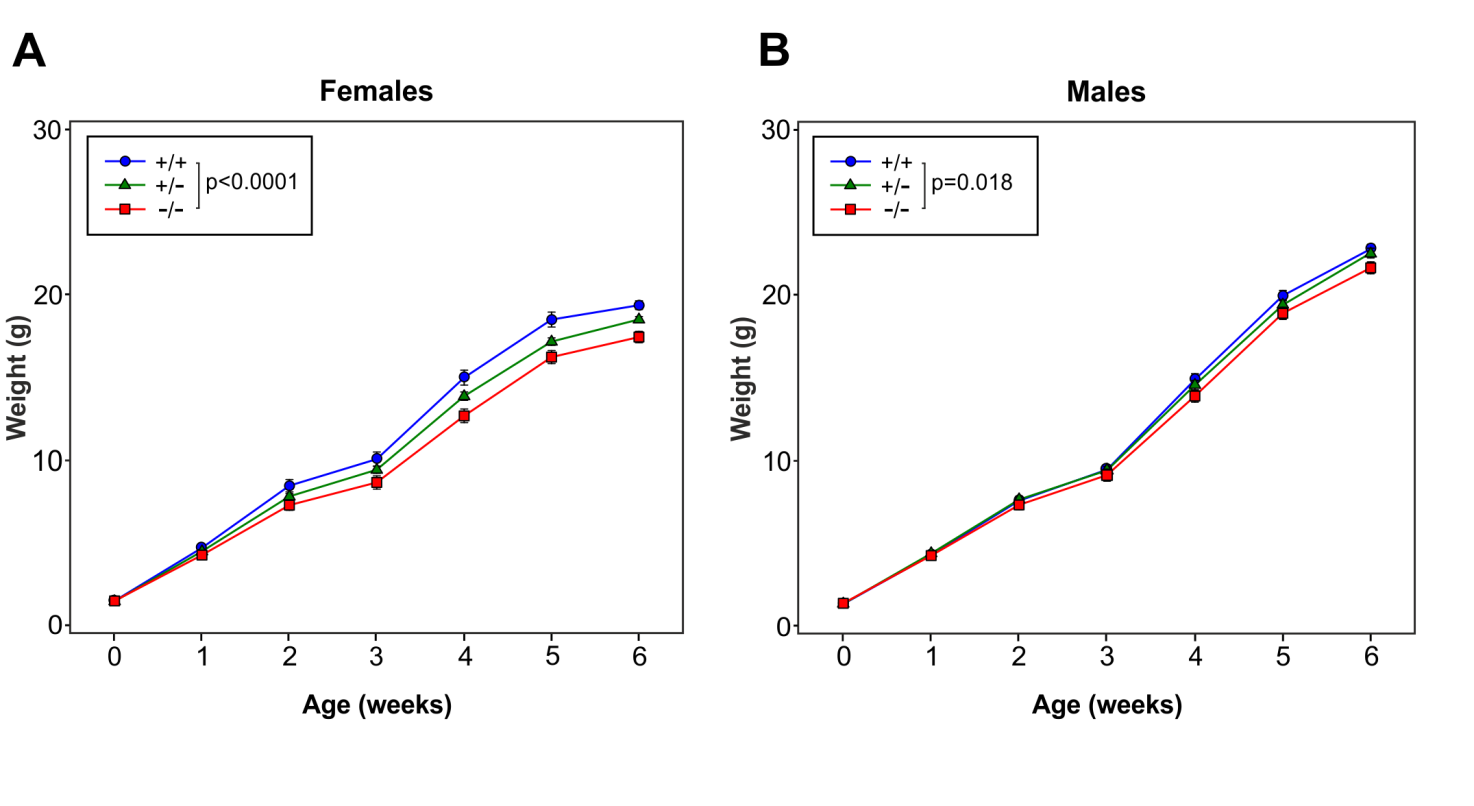
**

**Supplementary figure S1: The weight difference occurs shortly after birth**

A cohort of F3 female and male mice was weighed every week from the day at birth (day 0) until 6 weeks of age. Panel A shows the weight of the female mice [C4.4A^-/-^ (n=12), C4.4A^+/-^ (n=31) and C4.4A^+/+^ (n=10)] and panel B the weight of the male mice [C4.4A^-/-^ (n=11), C4.4A^+/-^ (n=33) and C4.4A^+/+^ (n=20)]. Both female and male C4.4A-deficient mice were significantly lighter than littermate controls. Standard errors of the mean are shown.

**
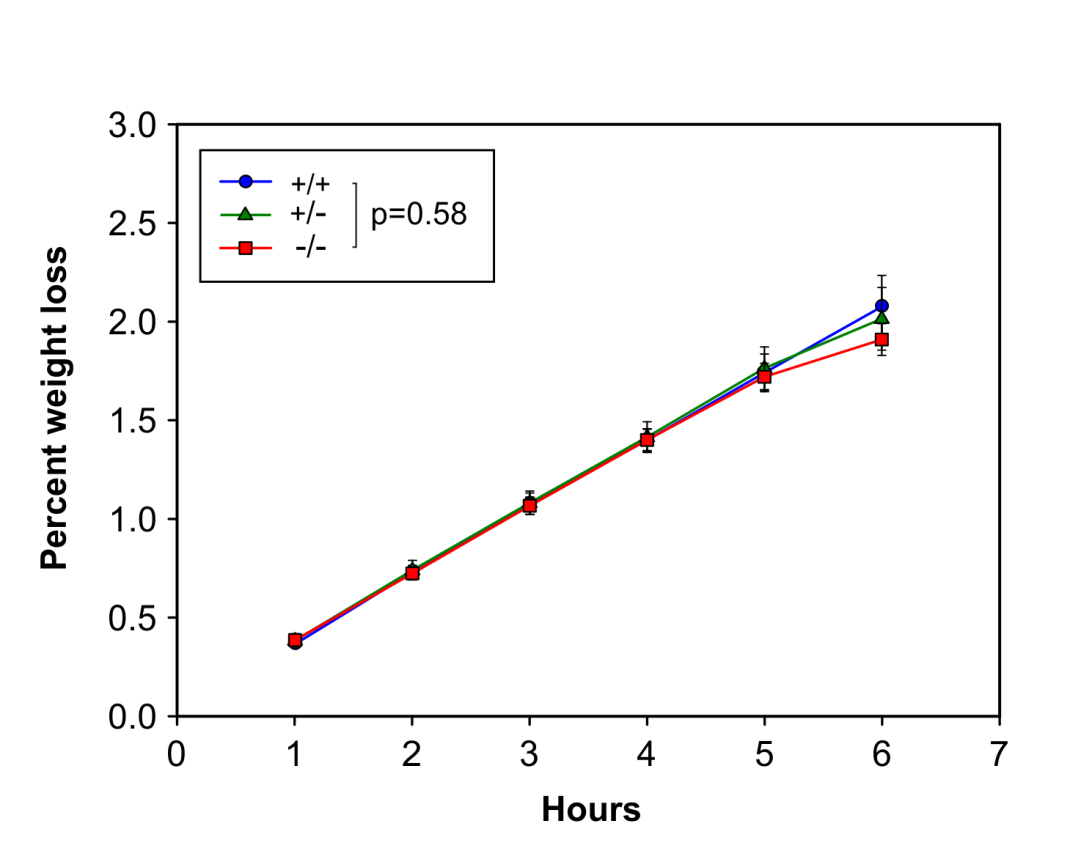
**

**Supplementary figure S2: C4.4A deficiency has no impact on skin barrier maintenance when analysed by TEWL**

Newborn littermate pups were incubated at 37°C for 6 hours and weighed every hour. The loss of transepidermal water was calculated as percentage loss of the total body weight. Pups that urinated were excluded during the experiment resulting in the following number of mice at the given time points: 0–2 hours: C4.4A^+/+^ n=17, C4.4A^+/-^ n=12, C4.4A^-/-^ n=12; 3 hours: C4.4A^+/+^: n=15, C4.4A^+/-^ n=12, C4.4A^-/-^ n=12; 4 hours: C4.4A^+/+^ n=14, C4.4A^+/-^ n=10, C4.4A^-/-^ n=12; 5 hours: C4.4A^+/+^ n=12, C4.4A^+/-^ n=9, C4.4A^-/-^ n=12; 6 hours: C4.4A^+/+^ n=8, C4.4A^+/-^ n=6, C4.4A^-/-^ n=7. Standard errors of the mean are shown.

**
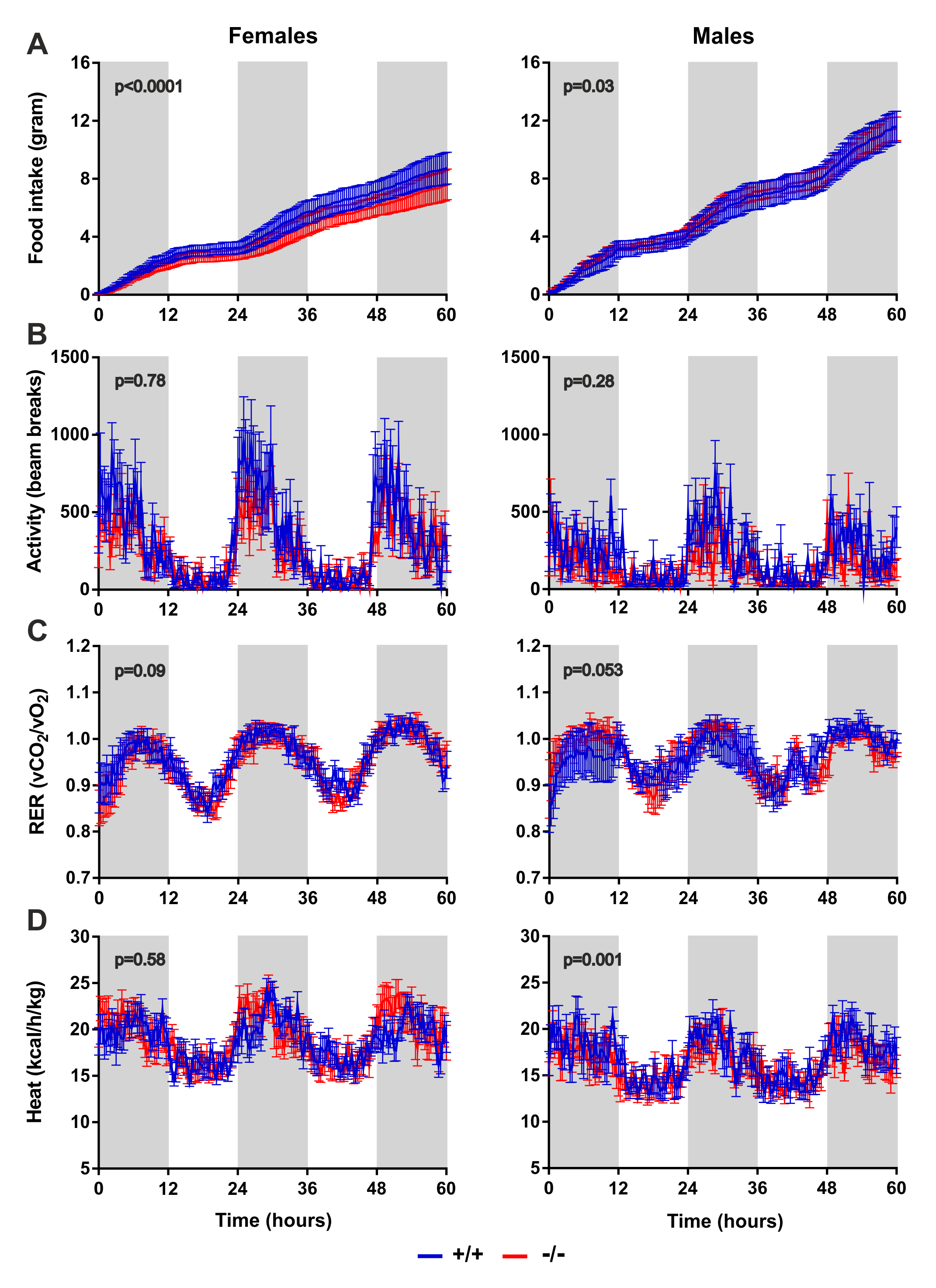
**

**Supplementary figure S3: Subtle effects of C4.4A deficiency on energy expenditures as assessed by metabolic cages**

12-14 weeks old C4.4A^-/-^ mice (females n=9, males n=5) and littermate control C4.4A^+/+^ (females n=8, males n=6) mice were individually housed for more than 7 days before simultaneously monitoring food intake (A), locomotion activity (B), respiratory exchange ratio (RER) (C) and energy expenditure (heat) (D). Data for 3 night (grey boxes) and 2 day (white boxes) phases are shown**.** Data were compared between genotypes for the entire observation period of 60 hours using mixed modeling with repeated measures. Due to the vast numbers of repeat measurements included in the statistical assessments subtle differences in the mean values become significant, but the biological significance of this observation is less clear, *e.g.* the 3 % difference increase in the energy expenditure of C4.4A ^+/+^ male mice. Standard errors of the mean are shown.

**
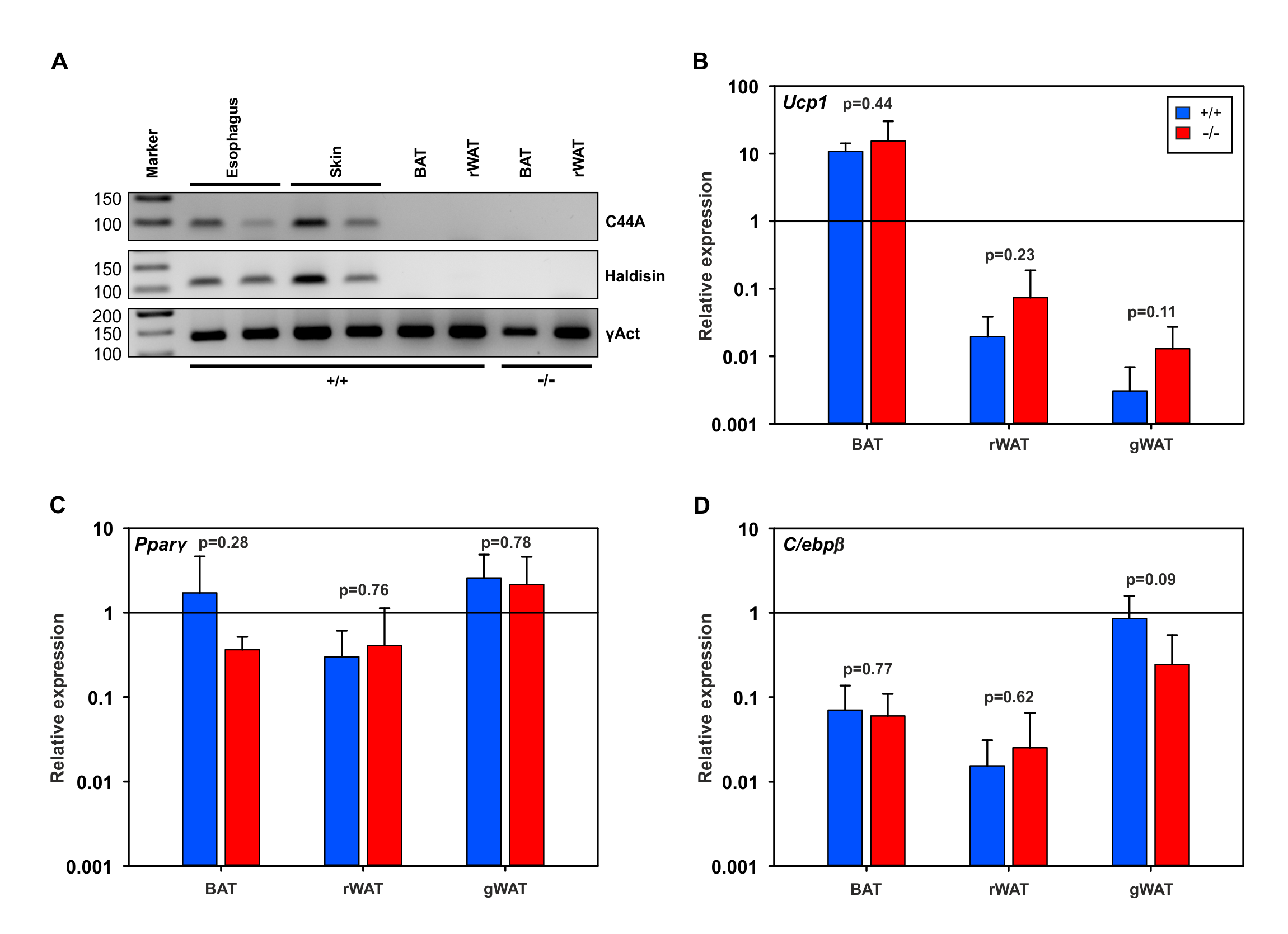
**

**Supplementary figure S4: Normal expression levels of selected genes in BAT and WAT from C4.4A-deficient mice**

Retroperitoneal and gonadal white adipose tissue depots (rWAT and gWAT) as well as brown adipose tissue depots (BAT) were resected from C4.4A^+/+^ and C4.4A^-/-^ mice, snap frozen in liquid N_2_, and RNA extracted. *Panel A*: Lack of mRNA expression of C4.4A and its homolog Haldisin in white and brown adipose tissues from C4.4A^+/+^ mice as determined by semi-quantitative PCR. Extracts from the squamous epithelia of esophagus and skin served as positive controls. mRNA expression levels of *Ucp1* (*panel B;* C4.4A^+/+^ n=7, C4.4A^-/-^ n=7), *Pparγ* (*panel C;* C4.4A^+/+^ n=5; C4.4A^-/-^ n=6 ) and *C/epbβ* (*panel D;* C4.4A^+/+^ n=5; C4.4A^-/-^ n=6) were quantified in rWAT, gWAT and BAT by qPCR. The relative expression levels represent data normalized to two stable housekeeping genes (*γAct* and *β_2_m*). Standard deviations are shown.

**
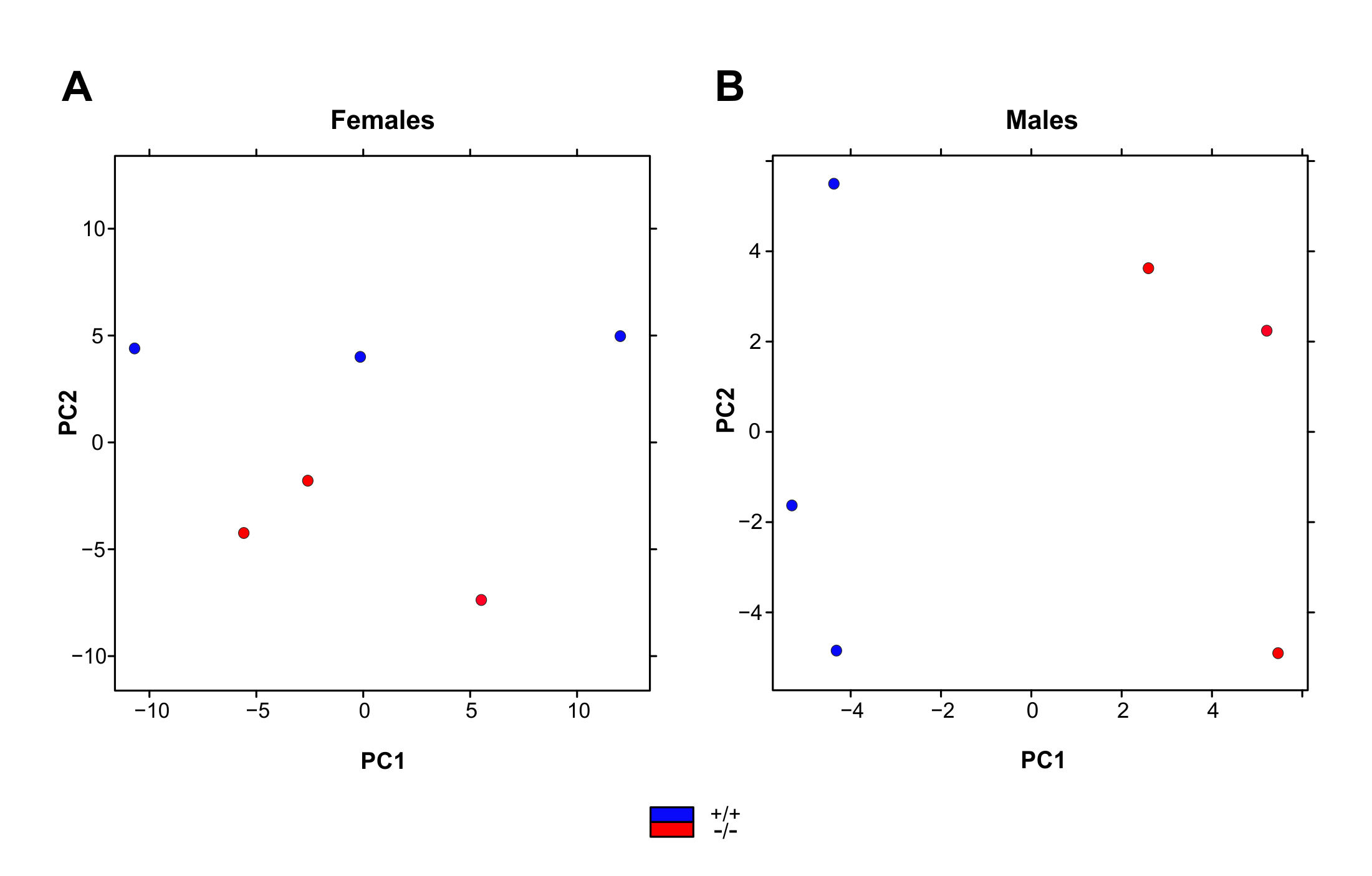
**

**Supplementary figure S5: Principal component analysis of the mRNA sequencing data**

The mRNA sequencing data from C4.4A^+/+^ and C4.4A^-/-^ female (A) and male (B) mice (3 of each), were evaluated by principal component analysis and plotted showing the first (PC1) and second (PC2) principal components.

**
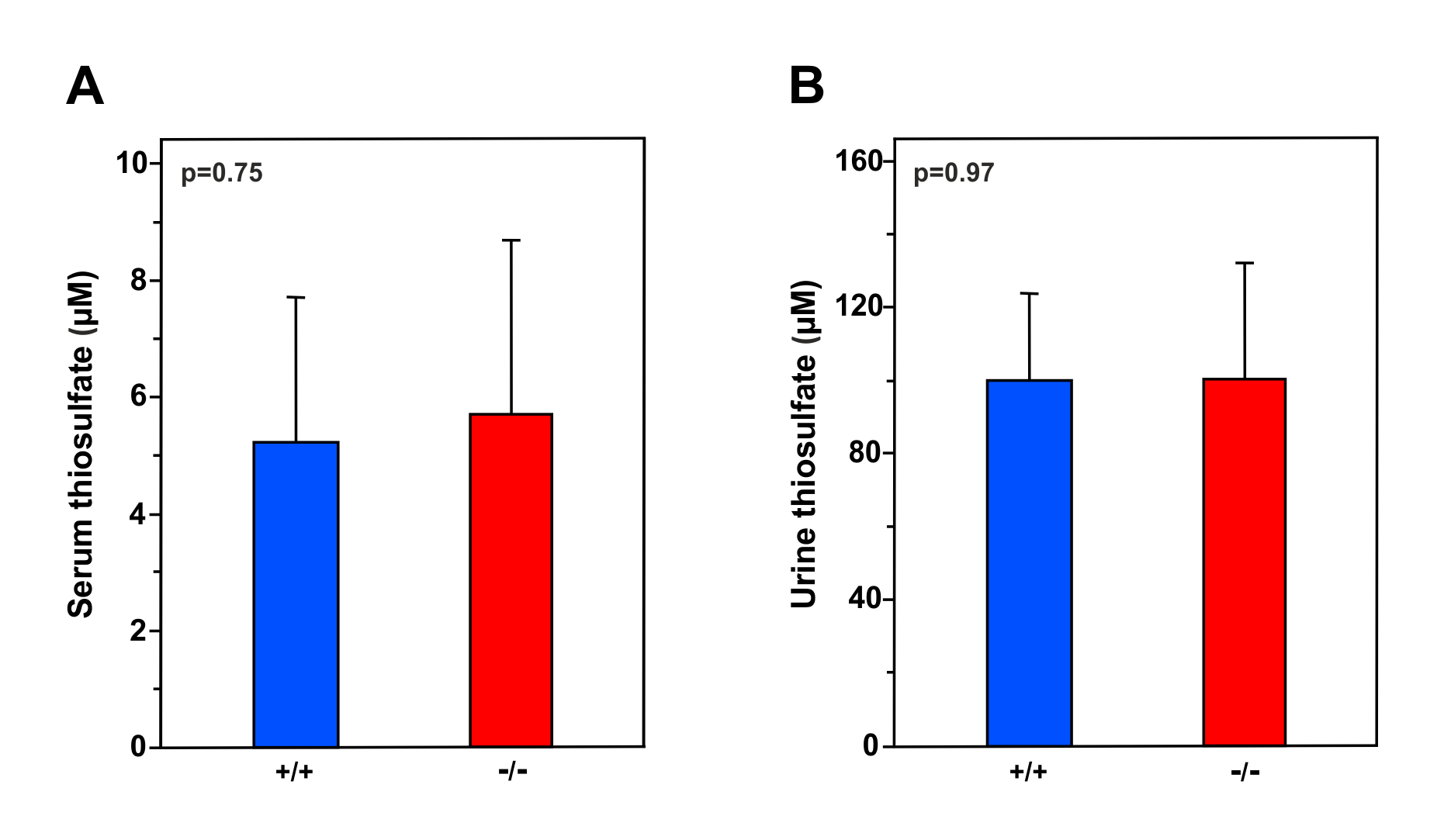
**

**Supplementary figure S6: The C4.4A-deficient mice have normal serum and urine thiosulfate levels**

The thiosulfate levels in serum (A) and urine (B) were measured in C4.4A^+/+^ (n=8) and C4.4A^-/-^ (n=8) mice. Note that the levels are within the lower limit of the normal ranges of 0–20 µM (serum) and 20–300 µM (urine)^6^. Standard deviations are shown.

**
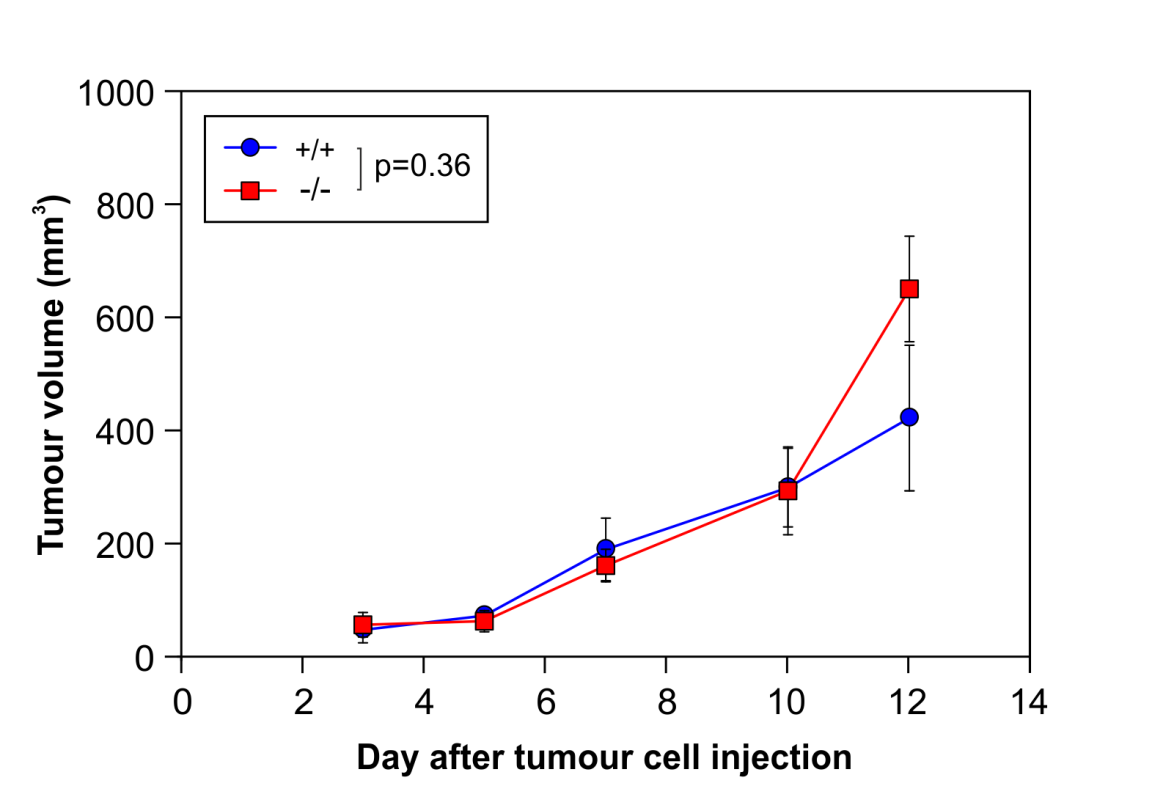
**

**Supplementary figure S7: C4.4A deficiency has no impact on the growth of engrafted Lewis Lung carcinoma cells**

Lewis Lung carcinoma cells were engrafted subcutaneously on the right flank of female C4.4A^+/+^ (n=5) and C4.4A^-/-^ (n=5) mice at day 0 and the tumour volume was measured using a calliper. Standard errors of the mean are shown.

**Supplemental references**

1 Völkel, S. & Grieshaber, M. K. Mechanisms of sulphide tolerance in the peanut worm, Sipunculus nudus (Sipunculidae) and in the lugworm, Arenicola marina (Polychaeta). *J. Comp. Physiol. B* **162**, 469-477 (1992).

2 Dobin, A. *et al.* STAR: ultrafast universal RNA-seq aligner. *Bioinformatics* **29**, 15-21 (2013).

3 Anders, S., Pyl, P. T. & Huber, W. HTSeq--a Python framework to work with high-throughput sequencing data. *Bioinformatics* **31**, 166-169 (2015).

4 Anders, S. & Huber, W. Differential expression analysis for sequence count data. *Genome Biol.* **11**, R106 (2010).

5 Solberg H., *et al*. A cleaved form of the receptor for urokinase-type plasminogen activator in invasive transplanted human and murine tumors. *Int J Cancer* **58**, 877-81 (1994).

6 Di Meo, I. *et al.* Effective AAV-mediated gene therapy in a mouse model of ethylmalonic encephalopathy. *EMBO Mol. Med.* **4**, 1008-1014 (2012).
